# Genome-Wide Association of Pericardial Fat Identifies a Unique Locus for Ectopic Fat

**DOI:** 10.1371/journal.pgen.1002705

**Published:** 2012-05-10

**Authors:** Caroline S. Fox, Charles C. White, Kurt Lohman, Nancy Heard-Costa, Paul Cohen, Yingying Zhang, Andrew D. Johnson, Valur Emilsson, Ching-Ti Liu, Y.-D. Ida Chen, Kent D. Taylor, Matthew Allison, Matthew Budoff, The CARDIoGRAM Consortium, Jerome I. Rotter, J. Jeffrey Carr, Udo Hoffmann, Jingzhong Ding, L. Adrienne Cupples, Yongmei Liu

**Affiliations:** 1 Framingham Heart Study, National Heart, Lung, and Blood Institute, Framingham, Massachusetts, United States of America; 2 Center for Population Studies, National Heart, Lung, and Blood Institute, Framingham, Massachusetts, United States of America; 3 Division of Endocrinology, Brigham and Women's Hospital and Harvard Medical School, Boston, Massachusetts, United States of America; 4 Department of Biostatistics, Boston University School of Public Health, Boston, Massachusetts, United States of America; 5 Department of Epidemiology and Prevention, Public Health Sciences, Wake Forest School of Medicine, Winston-Salem, North Carolina, United States of America; 6 Department of Neurology, Boston University School of Medicine, Boston, Massachusetts, United States of America; 7 Division of Cardiovascular Medicine and Department of Cancer Biology, Brigham and Women's Hospital and Dana Farber Cancer Institute, Boston, Massachusetts, United States of America; 8 Department of Cancer Biology, Dana Farber Cancer Institute, Boston, Massachusetts, United States of America; 9 Icelandic Heart Association, Hjartavernd, Iceland; 10 Medical Genetics Institute, Cedars-Sinai Medical Center, Los Angeles, California, United States of America; 11 Department of Preventive Medicine, University of California San Diego, La Jolla, California, United States of America; 12 Los Angeles Biomedical Research Institute, Torrance, California, United States of America; 13 Departments of Radiologic Sciences, Internal Medicine-Cardiology, and Public Health Sciences, Wake Forest University School of Medicine, Winston-Salem, North Carolina, United States of America; 14 Cardiac MR, PET, CT Program and the Department of Radiology, Massachusetts General Hospital, Boston, Massachusetts, United States of America; 15 Department of Internal Medicine/Geriatrics, Wake Forest School of Medicine, Winston-Salem, North Carolina, United States of America; Stanford University School of Medicine, United States of America

## Abstract

Pericardial fat is a localized fat depot associated with coronary artery calcium and myocardial infarction. We hypothesized that genetic loci would be associated with pericardial fat independent of other body fat depots. Pericardial fat was quantified in 5,487 individuals of European ancestry from the Framingham Heart Study (FHS) and the Multi-Ethnic Study of Atherosclerosis (MESA). Genotyping was performed using standard arrays and imputed to ∼2.5 million Hapmap SNPs. Each study performed a genome-wide association analysis of pericardial fat adjusted for age, sex, weight, and height. A weighted z-score meta-analysis was conducted, and validation was obtained in an additional 3,602 multi-ethnic individuals from the MESA study. We identified a genome-wide significant signal in our primary meta-analysis at rs10198628 near *TRIB2* (MAF 0.49, p = 2.7×10^-08^). This SNP was not associated with visceral fat (p = 0.17) or body mass index (p = 0.38), although we observed direction-consistent, nominal significance with visceral fat adjusted for BMI (p = 0.01) in the Framingham Heart Study. Our findings were robust among African ancestry (n = 1,442, p = 0.001), Hispanic (n = 1,399, p = 0.004), and Chinese (n = 761, p = 0.007) participants from the MESA study, with a combined p-value of 5.4E-14. We observed *TRIB2* gene expression in the pericardial fat of mice. rs10198628 near *TRIB2* is associated with pericardial fat but not measures of generalized or visceral adiposity, reinforcing the concept that there are unique genetic underpinnings to ectopic fat distribution.

## Author Summary

Pericardial fat is a localized fat depot associated with coronary artery calcium and myocardial infarction. To test whether genetic loci are associated with pericardial fat independent of other body fat depots, we measured pericardial fat in 5,487 individuals of European ancestry. After performing an unbiased screen using genome-wide association, we identified a genome-wide significant signal in our primary meta-analysis at rs10198628 near *TRIB2* (MAF 0.49, p = 2.7×10^-08^). This SNP was not associated with visceral fat (p = 0.17) or body mass index (p = 0.38). Our findings were robust among multi-ethnic participants from the MESA study, with a combined p-value of 5.4E-14. We observed *TRIB2* gene expression in the pericardial fat of mice. rs10198628 near *TRIB2* is associated with pericardial fat but not measures of generalized or visceral adiposity, reinforcing the concept that there are unique genetic underpinnings to ectopic fat distribution.

## Introduction

Obesity is a heterogeneous condition, and its attendant metabolic sequelae may not be adequately captured by using traditional metrics of generalized adiposity [1]. In part, this is because different fat depots may be associated with differential metabolic risk. For example, visceral abdominal fat is thought to be a unique pathogenic fat depot [2], [3].

Ectopic fat depots, defined as fat depots in non-classical locations [4], may mediate vascular disease due to their local toxic effect on nearby anatomic structures. We and others have shown that pericardial fat, defined as fat surrounding the heart and attendant structures, but not visceral fat, is associated with coronary artery calcification and coronary heart disease [5], [6]. The hypothesized local toxic effect of pericardial fat is supported by experimental research demonstrating perivascular inflammation [7] and smooth muscle cell proliferation [8].

Prior studies have shown that measures of generalized adiposity, including body mass index, are heritable [9]. In addition, more recent work has demonstrated that markers of body fat distribution, including waist-hip-ratio [10], subcutaneous and abdominal visceral fat [3], and fatty liver [11] also have a heritable component. Recent large scale genome-wide association studies (GWAS) have identified genomic loci for indices of body fat distribution that are independent of BMI [11]–[13], further supporting the concept that unique genetic variants exist that are associated with ectopic fat depots. To explore this further, we conducted a GWAS of pericardial fat to determine whether genetic loci are associated with the propensity to store fat around the heart.

## Results

### Study Sample Characteristics

The study sample characteristics are shown in Table 1 and Table S1. The mean age ranged from 55 years in the Framingham Heart Study to 62 years in MESA. In the MESA cohort, mean pericardial fat differed significantly between race/ethnicity groups. Compared to European Americans, mean pericardial fat was significantly lower in African Americans (P = 1.1E-38) and Chinese Americans (P = 4.8E-11), and was higher in Hispanic Americans (p = 0.02).

**Table 1:** Study sample characteristics.

Study	n	Women % (n)	Age (years)	BMI (kg/m2)	Pericardial Fat (cm3)
Framingham Heart Study	3100	48	55.4(11.8)	27.8(5.3)	113.6(44.7)
MESA study					
European Ancestry	2519	52 (1317)	62.7 (10.2)	27.7 (5.1)	85.3 (46.2)
African Ancestry	1609	54 (868)	62.3 (10.1)	30.1 (5.9)	68.0 (34.6)
Chinese	768	51 (390)	62.4 (10.4)	24.0 (3.3)	74.1 (31.6)
Hispanic	1445	52 (746)	61.4 (10.3)	29.4 (5.1)	88.6 (43.7)

Data shown as mean (standard deviation) unless otherwise indicated.

### Heritability Analyses and Genetic Correlations

The heritability (h2) of pericardial fat in the Framingham Heart Study was 50%. Upon additional adjustment for height and weight, the h2 was 52%.

We also calculated genetic correlations between pericardial fat, visceral fat (VAT), and BMI in the Framingham Heart Study. Genetic correlations between pericardial fat and VAT were 0.57; between pericardial fat and BMI 0.41, and between VAT and BMI 0.75. In all cases, we confirmed that there are genes that are associated with pair-wise comparisons of all three traits (all p-values<1.7*10E-15 for overlapping genetic correlations), although our results also suggest that not all genes are shared (all p-values<1.4*10E-22 for non-overlapping genetic correlations).

### GWAS Results of Meta-Analysis of the Framingham Heart Study and the MESA Study

The quantile-quantile plot (Figure S1) of GWAS of 5487 individuals of European ancestry demonstrated deviation from the null with no evidence of population stratification (lambda 0.99). The Manhattan plot (Figure S2) shows a genome-wide significant locus on chromosome 2 (p = 2.7E-08). The lead SNP (MAF 0.49) is rs10198628 located ∼80 kb upstream from the *TRIB2* gene. Per copy of the A allele, pericardial fat volumes were 4.4 cm^3^ lower in the Framingham Heart Study and 3.6 cm^3^ lower in MESA. All SNPs with p-values<1E-04 are shown in Table S2. We observed no evidence for a sex interaction for rs10198628 (p = 0.33). The variance of pericardial fat explained by the lead SNP in the Framingham Heart Study was 0.5% and 0.3% in MESA.

### Stage 2 Validation

**Figure 1:**
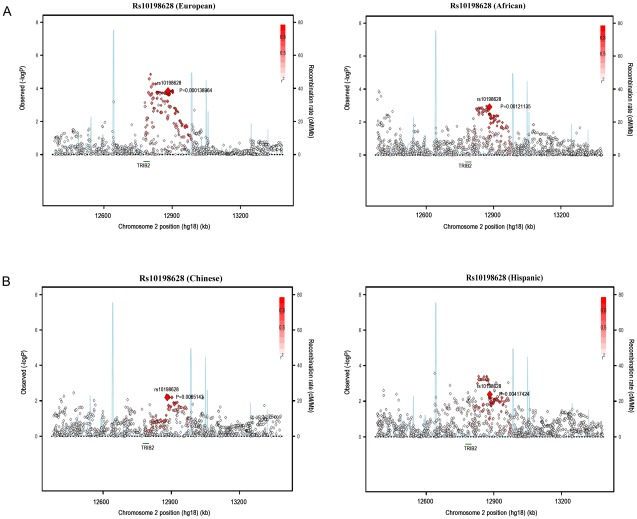
Regional association plots of rs10198628 in MESA ancestry populations. European, African (A), Chinese, and Hispanic ancestry population (B).

We performed validation in a multi-ethnic sample of African ancestry (n = 1442, β = 3.31, p = 0.001), Hispanic (n = 1399, β = 3.62, p = 0.004), and Chinese (n = 761, β = 4.56, p = 0.007) participants from the MESA study, with a combined Stage 1 and Stage 2 p-value of 5.4E-14 (Figure 1A and Figure 1B).

### Associations with Other Fat Depots

To assess whether rs10198628 is specific to pericardial fat, we assessed its associations with other fat depots (Table 2). We observed no association with body mass index from the GIANT consortium (p = 0.38) [14] or with visceral or subcutaneous fat from the Framingham Heart Study (p = 0.17 and 0.34, respectively). We observed nominal direction-consistent associations with waist-hip-ratio adjusted for body mass index from the GIANT consortium (p = 0.01) [12] and with visceral fat adjusted for body mass index in the Framingham Heart Study (p = 0.01).

**Table 2:** Results for rs10198628 across body composition and atherosclerosis traits in the Framingham Heart Study (n = 3,158) and the GIANT Consortium (n = 77,157 to 133,828) modeled per copy of the A allele.

Trait	Source	n	Beta*	P-value
Subcutaneous adipose tissue	FHS	3182	−0.025	0.34
Visceral adipose tissue	FHS	3158	−0.036	0.17
Visceral adipose tissue adj BMI	FHS	3146	−0.065	0.010
Visceral/Subcutaneous fat ratio	FHS	3158	−0.019	0.47
Body mass index	GIANT	123854	neg	0.38
Waist hip ratio adjusted BMI	GIANT	77157	neg	0.01
Height	GIANT	133828	neg	0.61
Carotid intimal medial thickness	MESA	2505	−0.051	0.04
Coronary Artery Calcium	MESA	2527	0.056	0.25
Coronary Heart Disease	CARDIoGRAM	819292	1.01	0.37

*: Odds ratio presented for CARDIoGRAM.

### Associations of Previously Published Loci for Body Fat Distribution and BMI with Pericardial Fat

We tested whether previously published SNPs in association with waist-hip-ratio adjusted for BMI [12] and BMI [14] are associated with pericardial fat in our meta-analysis dataset (Tables S4 and S5). Among the 14 well-validated SNPs for body fat distribution, we observed direction-consistent associations with *CPEB4*, a gene involved in cell survival [15]. Among SNPs associated with BMI, we observed nominal direction-consistent associations with *FTO*; no other associations were observed.

### Associations with Subclinical Atherosclerosis and Cardiovascular Disease Traits

Because of the proposed locally toxic effect of pericardial fat and cardiovascular disease outcomes, we evaluated the association of rs10198628 with several CVD phenotypes. We observed nominal, direction-consistent associations with carotid intimal medial thickness from the MESA individuals of European ancestry (n = 2505, p = 0.04), but we observed no association with myocardial infarction from the CARDIoGRAM consortium (p = 0.37, OR 1.01 [95% CI 0.985–1.04], n = 81929) [16] or with coronary artery calcification from the MESA study (p = 0.25, n = 2527).

### Association of Validated Coronary Heart Disease SNPs in the Pericardial Fat GWAS

We performed a look-up of 25 validated SNPs for coronary heart disease from the CARDIoGRAM consortium (Table S3) [16], and found that rs12190287 at *TCF21* was associated with pericardial fat in a direction-consistent fashion (p = 0.0019). No additional SNPs met the bonferroni corrected p-value threshold of p<0.002 (0.05/25).

### Gene Expression

We queried available human gene expression genetics data (see Methods) and identified eQTL associations with TRB2 in omental adipose (rs890069, 1.79e-9) [42] and two independent subcutaneous adipose samples (rs4669887, 3.62e-9; rs12616457, 1.28e-6) [41], [42]. All of these variants are in LD with our lead SNP rs10198628 (rs890069, rs4669887, rs1261657 have r2 0.38, 0.38 and 0.51 with rs10198629, respectively).

Next, we tested for gene expression in multiple subcutaneous (inguinal, axillary, and gluteal) and visceral (epididymal, retroperitoneal, mesenteric, omental) adipose depots as well as classical brown, pericardial, and perivascular adipose tissue which were dissected from high-fat fed male mice. mRNA expression levels of the adipocyte markers aP2 and PPARγ2 were comparable across all adipose depots. Trib2 was expressed in pericardial adipose tissue, as well as all of the other adipose depots surveyed (Figure S3). Expression in pericardial adipose tissue was comparable to that in other adipose depots. Lipin 1 is another annotated gene near our lead SNP. It was also expressed in pericardial adipose tissue, as well as all other depots surveyed. Furthermore, expression in pericardial adipose tissue was comparable to that in other adipose depots.

## Discussion

### Principal Findings

We have identified a SNP near the *TRIB2* locus that is associated with pericardial fat but not with body mass index or visceral abdominal fat. This SNP is also associated with pericardial fat in a multi-ethnic sample consisting of individuals of European, African, Hispanic, and Chinese ancestry. Finally, we identified a nearby eQTL, suggesting the potential for altered gene expression associated with our top SNP, or a correlated variant.

An important question of our work is whether rs10198628 is uniquely associated with pericardial fat, or merely represents a manifestation of generalized adiposity. Our results suggest that this SNP is unique in its association with pericardial fat, given its strong association with pericardial fat in our stage 1 and stage 2 analysis. In contrast, this SNP was not associated with visceral abdominal fat, an ectopic fat depot that is correlated with pericardial fat. Further, we observed no association with our lead SNP and body mass index in more than 100,000 individuals from the GIANT consortium. We note that we observed nominal significance with our lead SNP with VAT-adjusted-for-BMI and waist-hip-ratio-adjusted for BMI, traits representing fat distribution.

Gene expression analysis in mice showed that Trb2 is expressed in all adipose depots. Expression was not enriched in any depot. Confirmation that Trb2 is expressed in adipose tissue supports a functional role for this gene product in this tissue. Future studies are needed to investigate the specific function of Trb2 in adipose tissue, and in particular in pericardial fat. Finally, although our lead SNP is closest to Trb2, it is also possible that this is part of a regulatory region for another gene.

### In the Context of the Current Literature

Prior genome-wide association studies have primarily used easily-obtainable anthropometric measurements to estimate body fat distribution [12], [13]. While these studies benefit from concomitant enhanced power, the lack of detailed phenotyping renders the precise meaning of the measurement uncertain. In the current study, we have made use of well-validated measurements of pericardial fat that have been previously associated with coronary artery calcium [5], [17], myocardial infarction [6], [18], measurements of left ventricular structure and function [19], and carotid intimal medial thickness [20]. In this context, we sought to examine our lead SNP with coronary calcium, coronary heart disease, and carotid intimal medial thickness. Given the modest epidemiologic associations that have been observed in concert with the relatively small genetic effect sizes that are typical of GWAS, it is not surprising that we observed only nominal associations with carotid intimal medial thickness.

Our findings are also notable for some enrichment of association with a SNP in *TCF21*, previously identified in association with coronary heart disease in CARDIoGRAM [16]. *TCF21* encodes a transcription factor of the basic helix-loop-helix family and is a molecular marker of white adipose tissue [21]. *TCF21* is expressed in the epicardium of the developing zebrafish, and is associated with perivascular cells but not cardiomyocytes [22]. This is relevant given the anatomic location of pericardial fat and thus rendering it a form of perivascular fat surrounding the coronary arteries.

### Potential Mechanisms

Our lead SNP is located about 80 kb downstream from *TRIB2*. *TRIB2* is the tribbles homolog 2 gene, part of the Tribbles gene family. *TRIB2* expression has been shown to be elevated in lung cancer, and has been found to induce apoptosis through downregulation of the transcription factor CCAAT/enhancer-binding protein alpha (C/EBPα) [23]. Via this mechanism, *TRIB2* may also suppress adipocyte differentiation via AKt inhibition and C/EBPα degradation [24]. *TRIB2* has also been associated with hematologic abnormalities including acute myelogenous leukemia [25]. *TRIB2* has also been shown to be a regulator of the inflammatory activity of monocytes [26], suggesting a possible mechanism by which it may link low density lipoprotein cholesterol to plaque formation. *TRB1* and *TRB3* have both been linked to obesity and related phenotypes, with *TRIB1* gene expression linked to adipose tissue inflammation [27] and *TRB3* gene expression associated with insulin resistance [28].

Our lead SNP also lies near the *LPIN1* gene, a compelling candidate gene that has previously been identified in association with lipodystrophy syndromes [29]. While it is tempting to implicate this gene in our association analyses, it is notable that only rare, not common variants have been detected in human populations in association with lipodystrophic phenotypes [30]. In addition, our best SNP in the *LIPN1* gene has a p-value = 0.03, rendering it less likely. Finally, our lead SNP is ∼1 MB from *LPIN1*.

### Strengths and Limitations

Strengths of our study include the well-characterized pericardial fat data present in both the Framingham Heart Study and the MESA Study. An additional strength is the extension of our findings to multi-ethnic populations, underscoring the generalizability of this finding. Typical of GWAS, we have identified an associated locus, but the causal variant and gene remains unidentified. Limitations include the relatively modest sample size of our study, leading to relatively low power to detect small effects. While our finding that rs12190287 at *TCF21* was associated with pericardial fat in a direction-consistent fashion, we are unable to perform a formal mediation analysis.

### Implications

These findings support the concept that unique genetic variants exist in association with different ectopic fat depots. These findings are important because they suggest that different ectopic fat depots may each have their own unique genetic signature that is independent of generalized adiposity. Future work should focus on identifying the molecular mechanisms that link these genomic loci to ectopic fat depots, as this could ultimately lead to the identification of novel pathways and new therapeutic targets.

### Conclusions

A SNP near *TRIB2* is associated with pericardial fat but not measures of generalized or visceral adiposity, reinforcing the concept that there are unique genetic underpinnings to ectopic fat distribution.

## Methods

### Phenotype Definition

Pericardial fat was measured on CT using protocols determined by the participating studies, as described in the Study-Specific Methods. Sex-specific residuals were created, with adjustment for age, height, and weight, as well as principal components derived from genotypes denoting population stratification where necessary.

### Heritability Analyses

Heritability of pericardial fat was calculated in the Framingham Heart Study using standard methods. Sex-and-cohort specific residuals were created and then pooled for analysis using variance components analysis (SOLAR) [31].

### Genetic Correlations with Other Adiposity Traits

We used SOLAR [31] to calculate pair-wise genetic correlations between pericardial fat, visceral fat, and BMI in the Framingham Heart Study. We used residuals adjusted for age and sex. We tested two separate hypotheses: RhoG = 0 is the test for overlapping genetic correlations, whereas RhoG = 1 is the test for non-overlapping genetic correlations.

### Discovery Analyses

Table S1 and the study specific methods describe the genotyping that was conducted. Quality-control filters were used to exclude low-quality samples or SNPs. Each study imputed ∼2.5 million Phase 2 HapMap SNPs based on CEU samples; allelic dosage was used in the analysis.

Each cohort separately conducted the regression analysis, using an additive genetic effect model with accounting for family structure when necessary. Next, we conducted a fixed effects weighted Z-score meta-analysis given possible differences in phenotype scaling between the participating studies using METAL [32]. Statistical significance was considered when SNPs reached a meta-analysis *P* value≤5×10^−8^ [33]. Discovery analyses were performed on European ancestry participants. SNPs were filtered at a minor allele frequency<2% and an imputation quality score<0.3.

### Stage 2 Analysis

We conducted Stage 2 validation using non-white ethnic samples from the MESA study. Statistical significance was achieved when a direction-consistent p-value was at least p<0.05.

### Analyses of Related Phenotypes

For our lead SNP, we performed look-up in the publically available GIANT datasets [12], [14]. We also obtained specific look-up results in the CARDIoGRAM (for coronary heart disease) [16] and MESA (for coronary artery calcification and carotid intimal medial thickness).

We also tested whether the 25 previously-identified SNPs for coronary heart disease from the CARDIoGRAM consortium [16] were associated with pericardial fat. To determine statistical significance, we used the false discovery rate q-value; SNPs with q-value<0.05 were considered statistically significant using the QVALUE package in R [34].

### Interaction Testing

We tested for a formal sex interaction of rs10198628. Each study computed the interaction regression coefficient, standard error, and p-value. For the sex interaction, we included, age, height, weight, and any principal components (and study center) that were used in the original discovery analysis. We additionally added rs10198628 and the cross-product rs10198628*sex. Interaction terms were meta-analyzed using the weighted z-score approach.

### Variance Explained

We calculated the variance explained using the following formula: 2*MAF*(1−MAF)*(((beta)∧2)/((SD)∧2)).

### eSNP Analysis

We searched for eQTLs in a region bounded by the *LIPN1* and *TRIB2* genes using expression SNP (eSNP) datasets availably publically or via collaboration including lymphocytes [35], leukocytes [36], leukocytes from patients with Celiac disease [37], lymphoblastoid cell lines (LCL) from children with asthma [38], HapMap LCL from 3 populations [39], a separate study on HapMap CEU LCL [40], peripheral blood monocytes [41], [42], subcutaneous and omental adipose tissue [43], [44], and blood samples [43], 2 studies on brain cortex [41], [45], three large studies of brain regions including prefrontal cortex, visual cortex and cerebellum (Emilsson, personal communication), liver [44], [46], osteoblasts [47], skin [48], and additional fibroblast, T cell and LCL samples [49]. Statistical significance was considered using the association with gene transcript levels as originally described.

### Gene Expression

Adipose tissue was dissected from male C57Bl/6J mice (Jackson Labs) (n = 4) following 20 weeks of *ad libitum* feeding with a 60% high-fat diet (Research Diets, New Brunswick NJ). High fat feeding was used because many adipose depots (such as pericardial fat) are either not visible or extremely limited in size in standard chow fed mice. Animals were sacrificed and adipose tissues were dissected and frozen in liquid nitrogen. The following adipose depots were dissected: inguinal, axillary, gluteal, brown adipose, epididymal, retroperitoneal, mesenteric, omental, pericardial, and perivascular. All animal experiments were done according to procedures approved by the Institutional Animal Care and Use Committee of Beth Israel Deaconess Medical Center.

Total RNA was isolated using TRIzol (Invitrogen, Carlsbad, CA) combined with RNeasy mini-columns, (Qiagen, Valencia, CA) according to the manufacturer's instructions. For real-time PCR analysis, cDNA was synthesized from RNA using the high capacity cDNA synthesis kit (ABI, Carlsbad, CA). cDNA was used in quantitative PCR containing SYBR-green dye (ABI). mRNA expression levels for each gene were normalized to TBP. Quantitative PCR was performed using an ABI-7900HT PCR machine.

In addition to Lipin 1 and Trb2, the expression of the adipocyte markers aP2 and PPARγ2 were also measured. The primer sequences were as follows: aP2, forward 5′ CAT CAG CGT AAA TGG GGA TT 3′ reverse 5′ CCG CCA TCT AGG GTT ATG AT 3′; Lipin 1, forward 5′ CGT ACG TGC GGC TCT GCG AA 3′ reverse 5′ GCT CGG TCG CGT CAA GCT GA 3′; PPARγ2, forward 5′ GCA TGG TGC CTT CGC TGA 3′ reverse 5′ TGG CAT CTC TGT GTC AAC CAT G 3′; TBP, forward 5′ CCC CTT GTA CCC TTC ACC AAT 3′ reverse 5′ GAA GCT GCG GTA CAA TTC CAG 3′; Trib2, forward 5′ CCC GCC CGA GAC TCC GAA CT 3′ reverse 5′ GCA CAG CGC GGA AAA CGT GG 3′.

### Study-Specific Information

#### Framingham Heart Study

The Framingham Heart Study began in 1948 with the enrollment of the Original Cohort [50]. In 1971, the 5,124 participants were enrolled as part of the Offspring Cohort. Finally, in 2002, the Third Generation cohort was enrolled (n = 4095) [51]. Participants for the present study were derived from the Framingham Heart Study Multi-detector Computed Tomography (MDCT) Sub-Study. Briefly, from June 2002 to April 2005, 3529 participants (2111 Third Generation, 1418 Offspring participants) underwent MDCT.

Framingham Heart Study participants underwent MDCT utilizing 8-slice MDCT in a supine position (LightSpeed Ultra, General Electric, Milwaukee, WI). On average, 48 contiguous 2.5 mm slices of the heart were acquired with prospectively ECG triggered CT scanning protocol (120 kVp, 400 mA, temporal resolution 330 ms).

We measured pericardial fat tissue volumes (cm^3^) with a dedicated offline workstation (Aquarius 3D Workstation, TeraRecon Inc., San Mateo, CA) based on the principle that absolute Hounsfield Units (HU) values correspond to tissue property. Thus, we set a predefined image display (window width −195 to −45 HU; window center −120 HU) to identify pixels that correspond with adipose tissue. Pericardial fat was measured across the complete available imaging volume in cm^3^.

We used a semi-automatic segmentation technique which required the reader to manually trace the pericardium. We defined pericardial fat volume as adipose tissue located within the pericardial sac. Using a random sample of 100 participants, intra-reader (ICC 0.97) and inter-reader (ICC 0.95) reproducibility was excellent [5].

Framingham Heart Study participants also underwent eight-slice MDCT imaging of the abdomen with twenty-five contiguous five mm thick slices (120 kVp, 400 mA, gantry rotation time 500 ms, table feed 3∶1) acquired 125 mm beyond the level of S1. Subcutaneous and visceral adipose tissue volumes (SAT and VAT) were assessed as previously described, with excellent inter-class correlations for VAT (0.992) and SAT (0.997) [52].

The Phase II CEU HapMap panel was used a reference to impute genotypes to roughly 2.5 million HapMap SNPs; details can be found in Table S1. MACH v1.0.15/16 (http://www.sph.umich.edu/csg/abecasis/MACH/) was used along with 200 (101 Men and 99 Women) biologically independent individuals in order to establish parameter estimates. We then used these estimates to infer gene dosage. Imputed genotypes were expressed as allelic dosage (which is a decimal value ranging from 0–2).

Linear mixed effects regression modeling was used in order to account for pedigree structure (R lme and kinship package). Given that we observed association with the first principal component (estimated using Eigenstrat [53]), we included this component in our regression models.

#### MESA

The Multi-Ethnic Study of Atherosclerosis (MESA) is a National Heart, Lung and Blood Institute-sponsored, population-based investigation of subclinical cardiovascular disease and its progression [54]. In brief, a total of 6,809 individuals, aged 45 to 84 years, were recruited from six US communities (Baltimore City and County, MD; Chicago, IL; Forsyth County, NC; Los Angeles County, CA; New York, NY; and St. Paul, MN) between July 2000 and August 2002.

All MESA participants underwent baseline cardiac CT scans at baseline, which were analyzed for pericardial fat volume (cm3). Cardiac CT scans were performed either with an ECG-triggered (at 80% of the RR interval) electron-beam scanner (Chicago, Los Angeles, and New York field centers; Imatron C-150, Imatron) or with prospectively ECG-triggered scan acquisition at 50% of the RR interval with a multidetector system that acquired 4 simultaneous 2.5-mm slices for each cardiac cycle in a sequential or axial scan mode (Baltimore, Forsyth Country, and St. Paul field centers; Lightspeed, General Electric or Volume Zoom, Siemens). Three experienced CT analysts measured pericardial fat volume on the previously obtained images of the heart. For pericardial fat volume, slices within 15 mm above and 30 mm below the superior extent of the left main coronary artery were included. This region of the heart was selected because it includes the pericardial fat located around the proximal coronary arteries (left main coronary, left anterior descending, right coronary, and circumflex arteries). The anterior border of the volume was defined by the chest wall and the posterior border by the aorta and the bronchus. Volume Analysis software (GE Healthcare, Waukesha, WI) was used to discern fat from other tissues with a threshold of −190 to −30 Hounsfield units. The volume was the sum of all voxels containing fat. Our measure of pericardial fat volume was highly correlated with total volume of pericardial fat volume in a random subset of 10 Diabetes Heart Study participants (correlation coefficient: 0.93; p<0.0001). A random sample of 80 MESA participants was selected and their CT scans were reread. The intra-class correlation coefficients of intra-reader and inter-reader reliability were 0.99 and 0.89 for pericardial fat [6].

Coronary artery calcium was also assessed using the cardiac CT scans at baseline [55]. Briefly, we used the reader–work station interface to calibrate each tomographic image according to the estimated attenuation of the calcium phantom and then identified and quantified the coronary calcium in each image. We than calculated the coronary calcium score (Agatston score) for each scan. Intraobserver and interobserver agreement was excellent (kappa statistics, 0.93 and 0.90, respectively).

Carotid intimal medial thickness (IMT) measures were obtained at baseline using ultrasound imaging of the carotid arteries that was performed using a GE scanner. Using videotaped scans, we performed a centralized interpretation of the data. IMT was measured between lumen-intima and media-adventitia interfaces of near and far walls of the common carotid artery (the 1 cm segment proximal to the bifurcation) and the internal carotid artery (including the bifurcation and 1 cm distal to the bifurcation). A maximum IMT for each of these two segments was standardized (by subtraction of the MESA population mean and division by its standard deviation), and the mean of the standardized IMT for the common and the internal carotid maxima was used in analysis [56].

MESA participants provided consent for genotyping and had DNA extracted at the time of baseline enrollment between 2000–2002. Genotyping was performed at the Broad Institute of Harvard and MIT (Boston, Massachusetts, USA) and at the Affymetrix Laboratory (Santa Clara, CA, USA) using the Affymetric Genome-Wide Human SNP Array 6.0 (Affymetrix, Santa Clara, California, USA). Genotype results were imputed to 2.5 million SNPs using IMPUTE v.2.1.0 software (http://mathgen.stats.ox.ac.uk/impute/impute.html) [57]. Participant-specific quality controls included filters for call rate and number of Mendelian errors per individual. SNP-specific quality controls included filters for call rate and heterozygosity.

Linear regression modeling was used with adjustment for age, sex, weight, and height. The first principal component (estimated using Eigenstrat [53]) was included in our regression models.

## Supporting Information

Figure S1 Quantile–quantile plot.
(TIF)

Figure S2 Manhattan plot.
(TIF)

Figure S3 Mean normalized expression level and standard deviation for TRIB2 across a variety of fat depots.
(TIF)

Table S1 Genotyping information for each study.
(DOC)

Table S2 All SNPs with p-value<1E-04. Z-scores presented modeled per copy of Alelle1.
(DOC)

Table S3 Look-up of all validated Coronary Heart Disease SNPs as identified and validated by the CARDIoGRAM consortium with Pericardial Fat. Statistical significance derived from 25 independent tests (0.05/25 = 0.002).
(DOC)

Table S4 Look-up of SNPs from a Recently Published GWAS of Body Fat Distribution* (Heid IM et al, NG, 2010) [12]. All data modeled relative to the GIANT trait-increasing allele.
(DOC)

Table S5 Association of validated SNPs for BMI (from Speliotes et al, Nature Genetics 2010) [14]. All data modeled with the same coded allele in GIANT, and modeled relative to the GIANT trait-increasing allele.
(DOC)
