# Supplementary figures and images for: Genome-Wide Association of Pericardial Fat Identifies a Unique Locus for Ectopic Fat
Source: PLoS Genet. 2012 May 10;8(5):e1002705. doi: 10.1371/journal.pgen.1002705 (PMC3349742; doi:10.1371/journal.pgen.1002705)

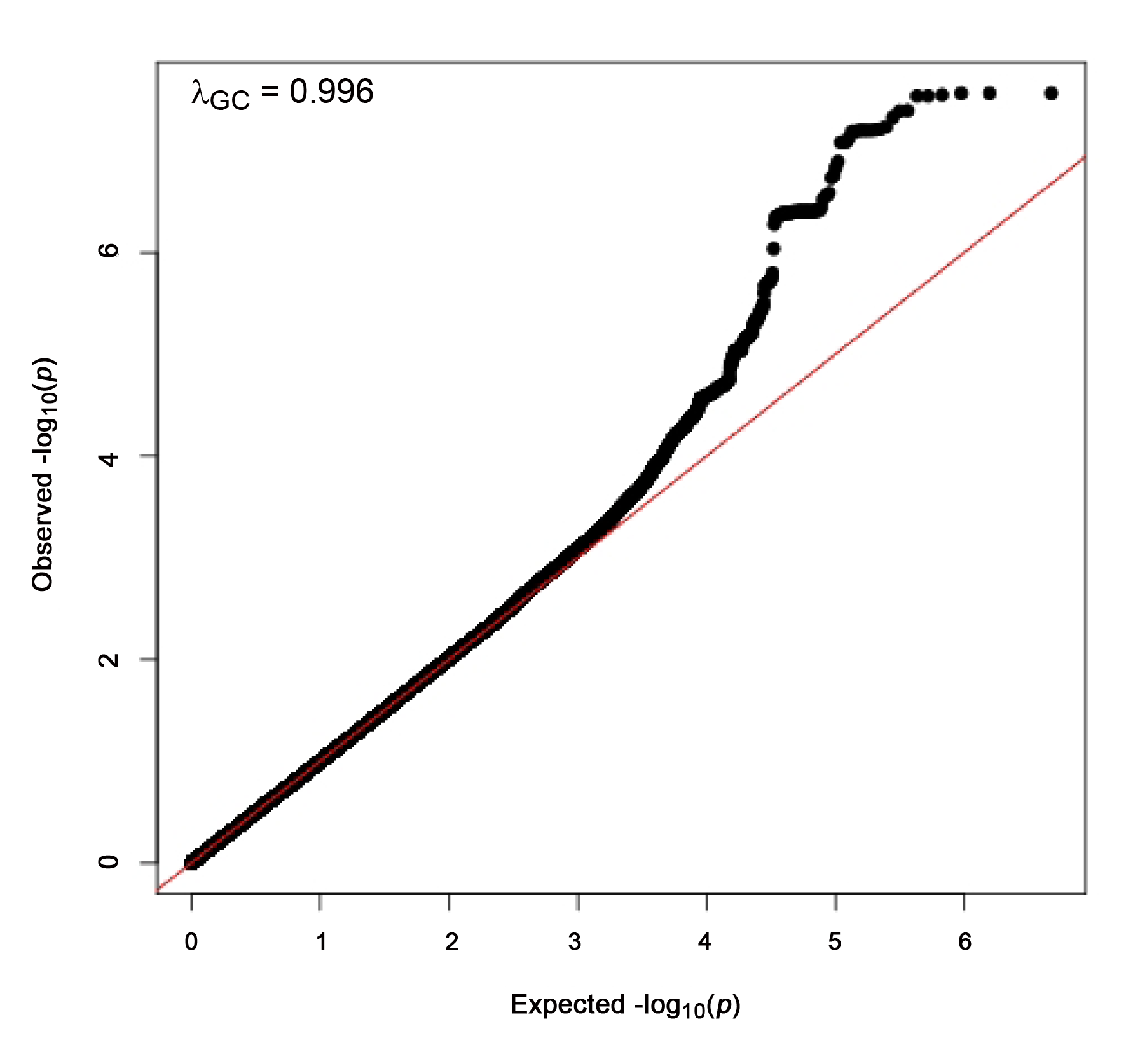

Supplement: Figure S1 — Quantile–quantile plot. (TIF) [file pgen.1002705.s001.tif]

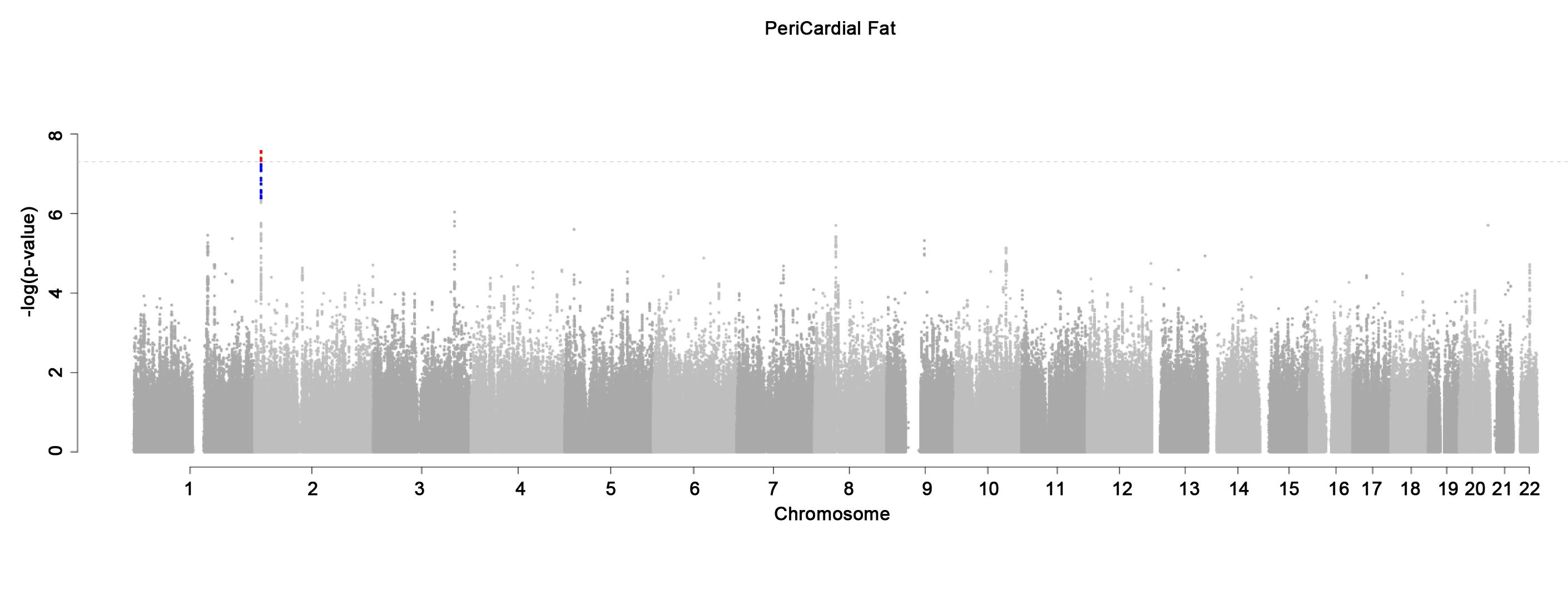

Supplement: Figure S2 — Manhattan plot. (TIF) [file pgen.1002705.s002.tif]

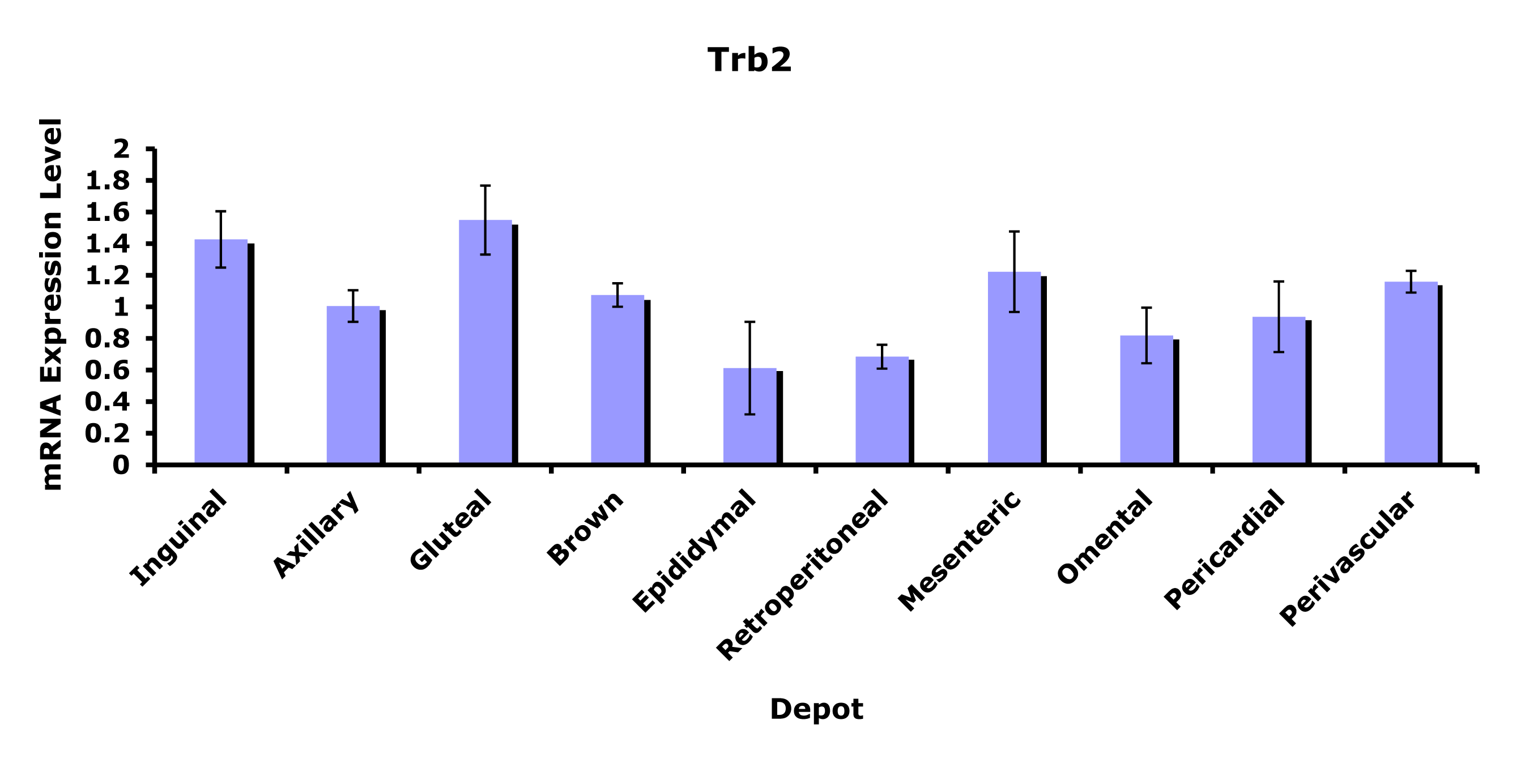

Supplement: Figure S3 — Mean normalized expression level and standard deviation for TRIB2 across a variety of fat depots. (TIF) [file pgen.1002705.s003.tif]
