# Supplementary material for: Genome-Wide Association of Pericardial Fat Identifies a Unique Locus for Ectopic Fat
Source: PLoS Genet. 2012 May 10;8(5):e1002705. doi: 10.1371/journal.pgen.1002705 (PMC3349742; doi:10.1371/journal.pgen.1002705)
Supplement: Table S2 — All SNPs with p-value<1E-04. Z-scores presented modeled per copy of Alelle1. (DOC) [file pgen.1002705.s005.doc]

| MarkerName | Chromosome | Location | Allele1 | Allele2 | Z-score | P.value | closestrefgene |
| --- | --- | --- | --- | --- | --- | --- | --- |
| rs10198628 | 2 | 12881948 | a | g | -5.56 | 2.7E-08 | TRIB2 |
| rs12621238 | 2 | 12881517 | t | c | -5.56 | 2.7E-08 | TRIB2 |
| rs10186351 | 2 | 12881879 | t | c | 5.56 | 2.7E-08 | TRIB2 |
| rs6432340 | 2 | 12880568 | t | c | -5.55 | 2.8E-08 | TRIB2 |
| rs10204312 | 2 | 12882864 | t | c | -5.55 | 2.9E-08 | TRIB2 |
| rs10192149 | 2 | 12880129 | a | g | -5.55 | 2.9E-08 | TRIB2 |
| rs6432341 | 2 | 12883265 | c | g | -5.49 | 4.0E-08 | TRIB2 |
| rs7572835 | 2 | 12883957 | a | g | 5.49 | 4.1E-08 | TRIB2 |
| rs10164960 | 2 | 12882610 | a | g | 5.46 | 4.7E-08 | TRIB2 |
| rs6732344 | 2 | 12901889 | a | g | 5.42 | 5.8E-08 | TRIB2 |
| rs4669869 | 2 | 12815911 | t | c | -5.41 | 6.2E-08 | TRIB2 |
| rs10929833 | 2 | 12902450 | t | c | -5.41 | 6.2E-08 | TRIB2 |
| rs890070 | 2 | 12902580 | t | g | 5.41 | 6.3E-08 | TRIB2 |
| rs4669885 | 2 | 12890470 | c | g | -5.41 | 6.3E-08 | TRIB2 |
| rs10184321 | 2 | 12887823 | a | c | -5.41 | 6.3E-08 | TRIB2 |
| rs12999821 | 2 | 12890991 | a | g | -5.41 | 6.3E-08 | TRIB2 |
| rs4669889 | 2 | 12898042 | t | c | -5.41 | 6.4E-08 | TRIB2 |
| rs12468536 | 2 | 12902413 | a | g | 5.38 | 7.5E-08 | TRIB2 |
| rs2098823 | 2 | 12885476 | a | c | 5.36 | 8.2E-08 | TRIB2 |
| rs890074 | 2 | 12827535 | a | g | 5.36 | 8.3E-08 | TRIB2 |
| rs890075 | 2 | 12827495 | t | c | -5.36 | 8.3E-08 | TRIB2 |
| rs7558818 | 2 | 12887423 | t | c | 5.28 | 1.3E-07 | TRIB2 |
| rs7558700 | 2 | 12887344 | c | g | -5.26 | 1.5E-07 | TRIB2 |
| rs7584581 | 2 | 12887438 | c | g | 5.22 | 1.8E-07 | TRIB2 |
| rs7559029 | 2 | 12887606 | a | c | 5.22 | 1.8E-07 | TRIB2 |
| rs1974923 | 2 | 12805153 | t | c | -5.15 | 2.6E-07 | TRIB2 |
| rs10179809 | 2 | 12880036 | a | c | 5.14 | 2.7E-07 | TRIB2 |
| rs10194305 | 2 | 12879816 | a | t | 5.14 | 2.8E-07 | TRIB2 |
| rs2113818 | 2 | 12808311 | t | c | -5.09 | 3.6E-07 | TRIB2 |
| rs6432331 | 2 | 12841562 | a | g | -5.08 | 3.8E-07 | TRIB2 |
| rs6432334 | 2 | 12846378 | t | g | -5.08 | 3.8E-07 | TRIB2 |
| rs1469217 | 2 | 12867575 | t | c | -5.07 | 3.9E-07 | TRIB2 |
| rs6730819 | 2 | 12868647 | t | c | 5.07 | 3.9E-07 | TRIB2 |
| rs1469218 | 2 | 12867543 | c | g | -5.07 | 3.9E-07 | TRIB2 |
| rs10175345 | 2 | 12868865 | t | g | -5.07 | 3.9E-07 | TRIB2 |
| rs732805 | 2 | 12866352 | a | g | 5.07 | 3.9E-07 | TRIB2 |
| rs2113815 | 2 | 12869156 | a | g | 5.07 | 3.9E-07 | TRIB2 |
| rs4583433 | 2 | 12867022 | t | g | 5.07 | 3.9E-07 | TRIB2 |
| rs12692486 | 2 | 12865597 | t | c | -5.07 | 3.9E-07 | TRIB2 |
| rs7580835 | 2 | 12856270 | a | g | 5.07 | 4.0E-07 | TRIB2 |
| rs10200958 | 2 | 12836666 | t | c | 5.07 | 4.0E-07 | TRIB2 |
| rs2113814 | 2 | 12869203 | t | c | 5.07 | 4.0E-07 | TRIB2 |
| rs2113813 | 2 | 12869218 | t | c | 5.07 | 4.0E-07 | TRIB2 |
| rs2113812 | 2 | 12869230 | a | g | -5.07 | 4.0E-07 | TRIB2 |
| rs6432336 | 2 | 12869401 | a | g | 5.07 | 4.0E-07 | TRIB2 |
| rs7597914 | 2 | 12869644 | a | g | -5.07 | 4.0E-07 | TRIB2 |
| rs2161996 | 2 | 12834994 | a | t | -5.07 | 4.1E-07 | TRIB2 |
| rs4668789 | 2 | 12871855 | a | g | 5.07 | 4.1E-07 | TRIB2 |
| rs13000123 | 2 | 12872277 | t | c | 5.07 | 4.1E-07 | TRIB2 |
| rs10929832 | 2 | 12873133 | t | c | 5.06 | 4.1E-07 | TRIB2 |
| rs1544853 | 2 | 12873221 | a | c | -5.06 | 4.1E-07 | TRIB2 |
| rs1544852 | 2 | 12873300 | t | c | -5.06 | 4.1E-07 | TRIB2 |
| rs1544851 | 2 | 12873349 | t | c | 5.06 | 4.1E-07 | TRIB2 |
| rs7598770 | 2 | 12873780 | t | c | -5.06 | 4.1E-07 | TRIB2 |
| rs1863176 | 2 | 12848543 | c | g | 5.06 | 4.2E-07 | TRIB2 |
| rs4669874 | 2 | 12832571 | t | c | -5.06 | 4.2E-07 | TRIB2 |
| rs4669873 | 2 | 12831992 | a | g | -5.06 | 4.2E-07 | TRIB2 |
| rs6751241 | 2 | 12874571 | t | c | -5.05 | 4.4E-07 | TRIB2 |
| rs7586442 | 2 | 12875071 | c | g | -5.05 | 4.4E-07 | TRIB2 |
| rs7586563 | 2 | 12875205 | t | c | 5.05 | 4.4E-07 | TRIB2 |
| rs4668792 | 2 | 12877261 | t | c | 5.05 | 4.5E-07 | TRIB2 |
| rs7581972 | 2 | 12834370 | t | c | -5.04 | 4.7E-07 | TRIB2 |
| rs2113811 | 2 | 12869945 | a | g | -5.02 | 5.3E-07 | TRIB2 |
| rs298751 | 3 | 165690414 | a | t | -4.91 | 9.2E-07 | SI |
| rs298755 | 3 | 165682048 | a | g | 4.80 | 1.6E-06 | SI |
| rs13011003 | 2 | 12818503 | a | c | 4.78 | 1.8E-06 | TRIB2 |
| rs7562714 | 2 | 12815833 | a | g | 4.76 | 1.9E-06 | TRIB2 |
| rs5022058 | 2 | 12814999 | c | g | -4.76 | 1.9E-06 | TRIB2 |
| rs2380453 | 2 | 12814742 | c | g | -4.76 | 1.9E-06 | TRIB2 |
| rs7267489 | 20 | 58404498 | t | c | 4.76 | 2.0E-06 | C20orf197 |
| rs7269082 | 20 | 58404569 | a | c | 4.76 | 2.0E-06 | C20orf197 |
| rs13256973 | 8 | 43259866 | t | c | 4.76 | 2.0E-06 | POTE8 |
| rs12616457 | 2 | 12814164 | a | g | 4.75 | 2.1E-06 | TRIB2 |
| rs298756 | 3 | 165682023 | a | g | -4.75 | 2.1E-06 | SI |
| rs1554443 | 2 | 12909195 | c | g | -4.74 | 2.2E-06 | TRIB2 |
| rs11957162 | 5 | 19310357 | a | g | 4.71 | 2.5E-06 | CDH18 |
| rs4669893 | 2 | 12915603 | c | g | 4.66 | 3.2E-06 | TRIB2 |
| rs7556962 | 2 | 12917491 | t | c | -4.65 | 3.3E-06 | TRIB2 |
| rs1995308 | 1 | 151280706 | a | t | 4.64 | 3.5E-06 | SPRR2D |
| rs1357060 | 2 | 12920449 | t | c | -4.64 | 3.6E-06 | TRIB2 |
| rs16891786 | 8 | 43261237 | t | g | -4.62 | 3.8E-06 | POTE8 |
| rs16875576 | 8 | 43260729 | t | c | 4.62 | 3.8E-06 | POTE8 |
| rs7007170 | 8 | 43256885 | a | c | -4.62 | 3.8E-06 | POTE8 |
| rs1521249 | 2 | 12926205 | t | c | -4.60 | 4.1E-06 | TRIB2 |
| rs7012007 | 8 | 43248604 | t | c | 4.60 | 4.2E-06 | POTE8 |
| rs2279127 | 1 | 201739564 | a | g | -4.60 | 4.3E-06 | OPTC |
| rs12623251 | 2 | 12868772 | t | c | 4.58 | 4.6E-06 | TRIB2 |
| rs6981146 | 8 | 43247170 | c | g | -4.58 | 4.7E-06 | POTE8 |
| rs17062170 | 9 | 77977853 | t | c | -4.58 | 4.8E-06 | PCSK5 |
| rs10188224 | 2 | 12913233 | t | g | -4.58 | 4.8E-06 | TRIB2 |
| rs2194724 | 2 | 12859493 | t | c | -4.57 | 4.9E-06 | TRIB2 |
| rs7015816 | 8 | 43246742 | t | c | -4.56 | 5.1E-06 | POTE8 |
| rs6474439 | 8 | 43269439 | a | g | 4.54 | 5.8E-06 | POTE8 |
| rs6474435 | 8 | 43253830 | a | t | -4.51 | 6.4E-06 | POTE8 |
| rs13250275 | 8 | 43262960 | t | c | -4.51 | 6.4E-06 | POTE8 |
| rs13254336 | 8 | 43260227 | t | g | -4.51 | 6.4E-06 | POTE8 |
| rs7000815 | 8 | 43259714 | a | c | 4.51 | 6.4E-06 | POTE8 |
| rs1108410 | 1 | 151272286 | t | c | -4.51 | 6.5E-06 | SPRR1B |
| rs12083211 | 1 | 151298755 | a | t | 4.50 | 6.7E-06 | SPRR2A |
| rs6664380 | 1 | 151296989 | t | g | 4.50 | 6.8E-06 | SPRR2A |
| rs12079087 | 1 | 151290512 | t | c | -4.50 | 6.9E-06 | SPRR2A |
| rs7008122 | 8 | 43248733 | a | c | 4.50 | 6.9E-06 | POTE8 |
| rs7012014 | 8 | 43248623 | t | c | 4.50 | 6.9E-06 | POTE8 |
| rs6587724 | 1 | 151301125 | a | t | 4.50 | 7.0E-06 | SPRR2A |
| rs6675009 | 1 | 151300407 | a | g | 4.50 | 7.0E-06 | SPRR2A |
| rs6988652 | 8 | 43248834 | t | c | -4.49 | 7.0E-06 | POTE8 |
| rs746552 | 10 | 104332491 | a | g | 4.48 | 7.4E-06 | SUFU |
| rs4669886 | 2 | 12891695 | a | g | -4.48 | 7.5E-06 | TRIB2 |
| rs7068535 | 10 | 104339117 | t | c | -4.48 | 7.5E-06 | SUFU |
| rs17062174 | 9 | 77978363 | a | c | -4.47 | 7.7E-06 | PCSK5 |
| rs4845513 | 1 | 151264690 | t | c | -4.47 | 7.8E-06 | SPRR1B |
| rs7531076 | 1 | 151305791 | t | g | 4.47 | 7.9E-06 | SPRR2B |
| rs10748827 | 10 | 104358326 | t | g | -4.46 | 8.2E-06 | SUFU |
| rs7016102 | 8 | 43264455 | a | g | -4.44 | 9.1E-06 | POTE8 |
| rs4855247 | 3 | 165679733 | t | c | -4.44 | 9.1E-06 | SI |
| rs6693927 | 1 | 151310633 | a | g | 4.44 | 9.1E-06 | SPRR2B |
| rs6686526 | 1 | 151311620 | t | c | -4.44 | 9.2E-06 | SPRR2B |
| rs6673356 | 1 | 151308527 | a | g | -4.44 | 9.2E-06 | SPRR2B |
| rs298760 | 3 | 165680450 | a | g | 4.44 | 9.2E-06 | SI |
| rs1500937 | 1 | 151312107 | c | g | 4.44 | 9.2E-06 | SPRR2B |
| rs298759 | 3 | 165680557 | c | g | 4.44 | 9.2E-06 | SI |
| rs7093285 | 10 | 104364805 | c | g | -4.44 | 9.2E-06 | SUFU |
| rs298758 | 3 | 165680860 | t | c | -4.44 | 9.2E-06 | SI |
| rs11812015 | 1 | 151312208 | t | g | 4.44 | 9.2E-06 | SPRR2B |
| rs11205181 | 1 | 151312352 | t | c | 4.44 | 9.2E-06 | SPRR2B |
| rs11205182 | 1 | 151312372 | t | c | 4.43 | 9.2E-06 | SPRR2B |
| rs11576797 | 1 | 151316041 | t | c | -4.43 | 9.2E-06 | SPRR2B |
| rs11590835 | 1 | 151313790 | t | c | -4.43 | 9.3E-06 | SPRR2B |
| rs7543689 | 1 | 151313427 | a | t | -4.43 | 9.3E-06 | SPRR2B |
| rs10888529 | 1 | 151317278 | t | g | 4.43 | 9.4E-06 | SPRR2B |
| rs11191347 | 10 | 104341607 | a | g | -4.42 | 9.9E-06 | SUFU |
| rs7013548 | 8 | 43242518 | a | g | 4.42 | 1.0E-05 | POTE8 |
| rs6692062 | 1 | 151318353 | t | c | -4.41 | 1.0E-05 | SPRR2B |
| rs10869720 | 9 | 77978293 | a | g | -4.41 | 1.0E-05 | PCSK5 |
| rs17062158 | 9 | 77976038 | a | c | 4.40 | 1.1E-05 | PCSK5 |
| rs7548977 | 1 | 151253989 | t | g | -4.40 | 1.1E-05 | SPRR3 |
| rs9521254 | 13 | 108695923 | t | c | 4.39 | 1.2E-05 | MYO16 |
| rs4669875 | 2 | 12833318 | c | g | 4.39 | 1.2E-05 | TRIB2 |
| rs2170206 | 8 | 43226253 | t | c | -4.38 | 1.2E-05 | POTE8 |
| rs13268355 | 8 | 43221720 | t | g | -4.37 | 1.3E-05 | HGSNAT |
| rs298757 | 3 | 165681444 | a | g | 4.37 | 1.3E-05 | SI |
| rs12617714 | 2 | 12888722 | a | g | -4.36 | 1.3E-05 | TRIB2 |
| rs2296588 | 10 | 104238077 | t | c | 4.32 | 1.6E-05 | ACTR1A |
| rs4669884 | 2 | 12886336 | t | c | 4.30 | 1.7E-05 | TRIB2 |
| rs12822389 | 12 | 130206849 | t | c | -4.29 | 1.8E-05 | GPR133 |
| rs11191330 | 10 | 104296150 | t | g | -4.29 | 1.8E-05 | SUFU |
| rs11191326 | 10 | 104292949 | t | c | -4.29 | 1.8E-05 | SUFU |
| rs11191325 | 10 | 104292923 | a | g | -4.28 | 1.8E-05 | SUFU |
| rs7096072 | 10 | 104289966 | t | c | -4.28 | 1.9E-05 | SUFU |
| rs298750 | 3 | 165692083 | t | c | 4.28 | 1.9E-05 | SI |
| rs298749 | 3 | 165693116 | c | g | -4.28 | 1.9E-05 | SI |
| rs12044569 | 1 | 165333420 | a | g | -4.27 | 1.9E-05 | DUSP27 |
| rs10748825 | 10 | 104283599 | a | g | 4.27 | 1.9E-05 | SUFU |
| rs5750124 | 22 | 34323096 | t | c | 4.27 | 1.9E-05 | MB |
| rs10918625 | 1 | 165330144 | a | g | 4.27 | 1.9E-05 | DUSP27 |
| rs10883732 | 10 | 104283451 | a | g | -4.27 | 1.9E-05 | SUFU |
| rs4234121 | 2 | 241417954 | t | g | 4.27 | 2.0E-05 | KIF1A |
| rs11191321 | 10 | 104283402 | t | c | -4.27 | 2.0E-05 | SUFU |
| rs10127745 | 1 | 165333366 | t | g | 4.26 | 2.0E-05 | DUSP27 |
| rs10918626 | 1 | 165332461 | t | g | 4.26 | 2.0E-05 | DUSP27 |
| rs6819856 | 4 | 93905918 | t | g | 4.26 | 2.0E-05 | GRID2 |
| rs7899004 | 10 | 104331425 | t | c | 4.26 | 2.0E-05 | SUFU |
| rs7907760 | 10 | 104335187 | t | c | -4.26 | 2.1E-05 | SUFU |
| rs7075269 | 10 | 104355714 | a | g | -4.26 | 2.1E-05 | SUFU |
| rs10786691 | 10 | 104354642 | a | g | -4.26 | 2.1E-05 | SUFU |
| rs10748826 | 10 | 104344794 | t | c | 4.26 | 2.1E-05 | SUFU |
| rs3934960 | 10 | 104345265 | a | g | -4.26 | 2.1E-05 | SUFU |
| rs11772378 | 7 | 95036561 | a | g | -4.26 | 2.1E-05 | PDK4 |
| rs12042641 | 1 | 165331649 | a | g | -4.25 | 2.1E-05 | DUSP27 |
| rs10883753 | 10 | 104362431 | t | c | -4.25 | 2.1E-05 | SUFU |
| rs11191352 | 10 | 104362234 | a | g | 4.25 | 2.1E-05 | SUFU |
| rs7908249 | 10 | 104363290 | a | g | 4.25 | 2.1E-05 | SUFU |
| rs729023 | 10 | 104331877 | t | c | -4.25 | 2.1E-05 | SUFU |
| rs17331190 | 1 | 165124587 | c | g | -4.25 | 2.1E-05 | TADA1L |
| rs12414407 | 10 | 104377009 | t | c | -4.25 | 2.1E-05 | SUFU |
| rs12784963 | 10 | 104326976 | a | g | 4.25 | 2.1E-05 | SUFU |
| rs10883736 | 10 | 104310019 | t | g | -4.25 | 2.2E-05 | SUFU |
| rs10786679 | 10 | 104305657 | a | g | -4.25 | 2.2E-05 | SUFU |
| rs4917973 | 10 | 104323690 | t | c | -4.25 | 2.2E-05 | SUFU |
| rs10127714 | 1 | 165333233 | a | c | 4.24 | 2.2E-05 | DUSP27 |
| rs4821421 | 22 | 34324229 | a | g | 4.24 | 2.2E-05 | MB |
| rs4917978 | 10 | 104356343 | t | c | 4.24 | 2.2E-05 | SUFU |
| rs3862030 | 10 | 104317574 | a | g | 4.24 | 2.3E-05 | SUFU |
| rs13274253 | 8 | 43227364 | t | g | -4.24 | 2.3E-05 | POTE8 |
| rs4917976 | 10 | 104350508 | t | c | -4.23 | 2.3E-05 | SUFU |
| rs2278117 | 2 | 12781221 | a | g | 4.23 | 2.3E-05 | TRIB2 |
| rs4919665 | 10 | 104356032 | t | c | 4.23 | 2.3E-05 | SUFU |
| rs10883738 | 10 | 104311711 | t | c | 4.23 | 2.3E-05 | SUFU |
| rs7907417 | 10 | 104360652 | t | c | -4.23 | 2.3E-05 | SUFU |
| rs4069493 | 2 | 97606235 | t | c | -4.23 | 2.3E-05 | COX5B |
| rs2296589 | 10 | 104238096 | a | t | -4.23 | 2.4E-05 | ACTR1A |
| rs7917158 | 10 | 104236461 | a | c | -4.23 | 2.4E-05 | ACTR1A |
| rs11590638 | 1 | 165337182 | c | g | -4.22 | 2.4E-05 | DUSP27 |
| rs10786680 | 10 | 104307182 | t | c | 4.22 | 2.4E-05 | SUFU |
| rs1056739 | 10 | 104226532 | t | c | 4.22 | 2.4E-05 | TMEM180 |
| rs2296583 | 10 | 104234312 | t | g | 4.22 | 2.4E-05 | ACTR1A |
| rs1056738 | 10 | 104226471 | a | g | -4.22 | 2.4E-05 | TMEM180 |
| rs10786696 | 10 | 104368124 | a | g | 4.22 | 2.4E-05 | SUFU |
| rs10883723 | 10 | 104215822 | t | c | 4.22 | 2.4E-05 | TMEM180 |
| rs2296586 | 10 | 104235565 | a | g | -4.22 | 2.4E-05 | ACTR1A |
| rs6584504 | 10 | 104243497 | t | c | 4.22 | 2.4E-05 | ACTR1A |
| rs7087984 | 10 | 104370676 | a | g | -4.22 | 2.5E-05 | SUFU |
| rs2296581 | 10 | 104232876 | a | g | -4.22 | 2.5E-05 | ACTR1A |
| rs11685678 | 2 | 12870715 | a | g | 4.22 | 2.5E-05 | TRIB2 |
| rs810692 | 3 | 165655524 | a | g | -4.21 | 2.5E-05 | SI |
| rs7074343 | 10 | 104273155 | a | g | -4.21 | 2.5E-05 | SUFU |
| rs7087665 | 10 | 104272408 | t | c | 4.21 | 2.5E-05 | SUFU |
| rs1409310 | 10 | 104272255 | t | c | 4.21 | 2.5E-05 | SUFU |
| rs1409311 | 10 | 104272150 | a | g | -4.21 | 2.5E-05 | SUFU |
| rs11594073 | 10 | 104298183 | t | c | -4.21 | 2.5E-05 | SUFU |
| rs6584508 | 10 | 104248557 | a | g | 4.21 | 2.6E-05 | ACTR1A |
| rs7090034 | 10 | 104271145 | c | g | 4.21 | 2.6E-05 | SUFU |
| rs10786673 | 10 | 104270527 | a | g | -4.21 | 2.6E-05 | SUFU |
| rs2296590 | 10 | 104252618 | a | g | -4.21 | 2.6E-05 | ACTR1A |
| rs17465771 | 2 | 12933619 | c | g | 4.21 | 2.6E-05 | TRIB2 |
| rs3808934 | 10 | 104254270 | t | c | -4.21 | 2.6E-05 | SUFU |
| rs10786684 | 10 | 104313018 | t | g | -4.21 | 2.6E-05 | SUFU |
| rs2296579 | 10 | 104231365 | a | g | 4.21 | 2.6E-05 | ACTR1A |
| rs5750125 | 22 | 34326244 | a | t | -4.21 | 2.6E-05 | MB |
| rs13136254 | 4 | 185061878 | t | c | -4.21 | 2.6E-05 | STOX2 |
| rs11191312 | 10 | 104268591 | t | c | 4.21 | 2.6E-05 | SUFU |
| rs9597065 | 13 | 54440324 | a | g | 4.20 | 2.6E-05 | OLFM4 |
| rs2296582 | 10 | 104234129 | t | g | -4.20 | 2.6E-05 | ACTR1A |
| rs13287 | 10 | 104230428 | a | g | 4.20 | 2.7E-05 | ACTR1A |
| rs2309166 | 2 | 97605918 | a | c | 4.20 | 2.7E-05 | COX5B |
| rs1884929 | 10 | 104265408 | t | c | -4.20 | 2.7E-05 | SUFU |
| rs4821422 | 22 | 34330493 | a | g | 4.20 | 2.7E-05 | MB |
| rs10200527 | 2 | 12851368 | t | c | -4.20 | 2.7E-05 | TRIB2 |
| rs4820205 | 22 | 34331204 | t | c | 4.20 | 2.7E-05 | MB |
| rs10883728 | 10 | 104259291 | a | g | -4.20 | 2.7E-05 | SUFU |
| rs2281879 | 10 | 104258867 | c | g | -4.20 | 2.7E-05 | SUFU |
| rs7094366 | 10 | 104264720 | c | g | 4.20 | 2.7E-05 | SUFU |
| rs10786671 | 10 | 104261293 | a | g | -4.20 | 2.7E-05 | SUFU |
| rs10883729 | 10 | 104263933 | a | g | 4.20 | 2.7E-05 | SUFU |
| rs4269466 | 7 | 95028689 | t | c | 4.20 | 2.7E-05 | PDK4 |
| rs1056857 | 10 | 104229993 | t | c | -4.19 | 2.8E-05 | ACTR1A |
| rs10786682 | 10 | 104308312 | t | g | -4.18 | 2.9E-05 | SUFU |
| rs6552716 | 4 | 185060940 | a | t | 4.18 | 2.9E-05 | STOX2 |
| rs7095433 | 10 | 72728378 | a | t | -4.18 | 2.9E-05 | UNC5B |
| rs247207 | 5 | 128113341 | t | c | 4.18 | 2.9E-05 | FBN2 |
| rs2922564 | 2 | 97518907 | t | c | 4.18 | 3.0E-05 | KIAA1641 |
| rs12499252 | 4 | 126190738 | t | c | -4.17 | 3.0E-05 | FAT4 |
| rs4140603 | 22 | 34331515 | t | c | 4.17 | 3.1E-05 | MB |
| rs9610322 | 22 | 34310119 | a | g | -4.16 | 3.2E-05 | MB |
| rs16944011 | 18 | 23546361 | c | g | 4.15 | 3.3E-05 | CDH2 |
| rs4669872 | 2 | 12818933 | c | g | 4.15 | 3.3E-05 | TRIB2 |
| rs4669887 | 2 | 12893630 | t | g | -4.15 | 3.3E-05 | TRIB2 |
| rs973977 | 2 | 12822941 | t | c | 4.15 | 3.3E-05 | TRIB2 |
| rs6432342 | 2 | 12939711 | a | g | -4.14 | 3.5E-05 | TRIB2 |
| rs3906948 | 2 | 97591869 | a | g | -4.14 | 3.5E-05 | KIAA1641 |
| rs4286667 | 5 | 19269375 | c | g | -4.14 | 3.5E-05 | CDH18 |
| rs4669876 | 2 | 12835751 | t | c | 4.14 | 3.5E-05 | TRIB2 |
| rs10236474 | 7 | 95053313 | t | g | 4.14 | 3.6E-05 | PDK4 |
| rs4285804 | 10 | 104376299 | a | t | 4.14 | 3.6E-05 | SUFU |
| rs4069494 | 2 | 97606236 | a | g | 4.13 | 3.6E-05 | COX5B |
| rs1546350 | 17 | 28781624 | a | g | 4.13 | 3.7E-05 | ACCN1 |
| rs12712056 | 2 | 97536554 | t | c | -4.13 | 3.7E-05 | KIAA1641 |
| rs9368182 | 6 | 20451410 | t | g | -4.12 | 3.7E-05 | E2F3 |
| rs2342456 | 4 | 61806648 | a | c | -4.12 | 3.8E-05 | LPHN3 |
| rs1965204 | 2 | 97506481 | a | g | -4.12 | 3.8E-05 | KIAA1641 |
| rs662506 | 1 | 151315010 | a | c | 4.12 | 3.8E-05 | SPRR2B |
| rs11691779 | 2 | 97533360 | a | c | 4.12 | 3.9E-05 | KIAA1641 |
| rs11690658 | 2 | 97529635 | t | g | 4.12 | 3.9E-05 | KIAA1641 |
| rs6718518 | 2 | 241426555 | t | c | 4.12 | 3.9E-05 | KIF1A |
| rs956596 | 2 | 12945111 | a | g | -4.11 | 3.9E-05 | TRIB2 |
| rs4676347 | 2 | 241419616 | a | t | 4.11 | 3.9E-05 | KIF1A |
| rs13001551 | 2 | 97540866 | a | t | 4.11 | 3.9E-05 | KIAA1641 |
| rs3931133 | 2 | 97542772 | a | g | 4.11 | 3.9E-05 | KIAA1641 |
| rs10489574 | 1 | 165177455 | a | g | -4.11 | 3.9E-05 | C1orf32 |
| rs17773812 | 14 | 88832561 | a | g | 4.11 | 4.0E-05 | FOXN3 |
| rs10198180 | 2 | 12853641 | a | g | 4.11 | 4.0E-05 | TRIB2 |
| rs9284729 | 2 | 34090505 | t | g | 4.11 | 4.0E-05 | FAM98A |
| rs6718109 | 2 | 97542295 | t | c | -4.11 | 4.0E-05 | KIAA1641 |
| rs407448 | 1 | 151274128 | t | c | 4.11 | 4.0E-05 | SPRR1B |
| rs6727552 | 2 | 12849758 | t | g | 4.10 | 4.1E-05 | TRIB2 |
| rs7221119 | 17 | 28797391 | a | g | -4.10 | 4.1E-05 | ACCN1 |
| rs13266490 | 8 | 43203562 | t | c | -4.10 | 4.2E-05 | HGSNAT |
| rs12994535 | 2 | 97575123 | t | g | 4.10 | 4.2E-05 | KIAA1641 |
| rs4821423 | 22 | 34331108 | t | c | 4.10 | 4.2E-05 | MB |
| rs2073983 | 7 | 95056673 | a | g | 4.10 | 4.2E-05 | PDK4 |
| rs821755 | 1 | 151302869 | c | g | 4.09 | 4.3E-05 | SPRR2A |
| rs608509 | 1 | 151301274 | c | g | 4.09 | 4.3E-05 | SPRR2A |
| rs12512668 | 4 | 126183777 | t | g | -4.09 | 4.3E-05 | FAT4 |
| rs11585767 | 1 | 151408952 | t | c | 4.09 | 4.4E-05 | SPRR2G |
| rs582345 | 1 | 151300030 | a | g | 4.09 | 4.4E-05 | SPRR2A |
| rs2059427 | 2 | 12811428 | t | c | 4.09 | 4.4E-05 | TRIB2 |
| rs247168 | 5 | 128096024 | a | g | 4.09 | 4.4E-05 | FBN2 |
| rs387930 | 1 | 151316682 | a | g | -4.09 | 4.4E-05 | SPRR2B |
| rs399550 | 1 | 151316541 | t | c | -4.09 | 4.4E-05 | SPRR2B |
| rs2261596 | 2 | 97587482 | a | g | -4.09 | 4.4E-05 | KIAA1641 |
| rs10846018 | 12 | 7876585 | a | c | 4.08 | 4.4E-05 | SLC2A14 |
| rs2442166 | 2 | 97587458 | a | c | -4.08 | 4.4E-05 | KIAA1641 |
| rs310126 | 1 | 151286040 | a | c | -4.08 | 4.5E-05 | SPRR2D |
| rs12668651 | 7 | 95056374 | t | c | -4.08 | 4.5E-05 | PDK4 |
| rs6868154 | 5 | 19276744 | a | g | -4.08 | 4.5E-05 | CDH18 |
| rs6747537 | 2 | 12917848 | t | c | 4.08 | 4.6E-05 | TRIB2 |
| rs440598 | 1 | 151311357 | t | c | -4.08 | 4.6E-05 | SPRR2B |
| rs1048296 | 1 | 151278864 | a | c | -4.07 | 4.7E-05 | SPRR2D |
| rs1750309 | 1 | 151280254 | c | g | 4.07 | 4.7E-05 | SPRR2D |
| rs1521246 | 2 | 12955369 | a | c | -4.07 | 4.7E-05 | TRIB2 |
| rs10788856 | 1 | 151278141 | c | g | 4.07 | 4.7E-05 | SPRR2D |
| rs4069489 | 2 | 97606081 | a | g | -4.07 | 4.7E-05 | COX5B |
| rs561143 | 1 | 151277358 | t | c | 4.07 | 4.7E-05 | SPRR2D |
| rs409986 | 1 | 151281365 | a | c | 4.07 | 4.8E-05 | SPRR2D |
| rs406350 | 1 | 151274116 | t | c | 4.07 | 4.8E-05 | SPRR1B |
| rs557553 | 1 | 151276994 | t | g | -4.07 | 4.8E-05 | SPRR2D |
| rs451939 | 1 | 151273501 | t | c | 4.06 | 4.8E-05 | SPRR1B |
| rs6681328 | 1 | 201671258 | a | g | 4.06 | 4.9E-05 | PRELP |
| rs382292 | 1 | 151273265 | a | g | 4.06 | 4.9E-05 | SPRR1B |
| rs489323 | 1 | 151272671 | a | g | 4.06 | 4.9E-05 | SPRR1B |
| rs12357172 | 10 | 104378539 | t | c | -4.06 | 5.0E-05 | SUFU |
| rs423692 | 1 | 151272305 | t | g | -4.06 | 5.0E-05 | SPRR1B |
| rs11578865 | 1 | 151392926 | a | g | 4.05 | 5.0E-05 | SPRR2G |
| rs1334848 | 1 | 151271345 | a | t | -4.05 | 5.1E-05 | SPRR1B |
| rs310105 | 1 | 151288877 | a | g | -4.05 | 5.1E-05 | SPRR2A |
| rs1334850 | 1 | 151271143 | t | g | 4.05 | 5.1E-05 | SPRR1B |
| rs10472930 | 5 | 19287563 | t | g | 4.05 | 5.1E-05 | CDH18 |
| rs2070964 | 1 | 151270837 | t | c | 4.05 | 5.2E-05 | SPRR1B |
| rs4845519 | 1 | 151270540 | a | c | -4.05 | 5.2E-05 | SPRR1B |
| rs1019136 | 5 | 128091175 | a | g | 4.05 | 5.2E-05 | FBN2 |
| rs779339 | 2 | 12961335 | a | g | 4.05 | 5.2E-05 | TRIB2 |
| rs4845518 | 1 | 151270527 | a | g | -4.05 | 5.2E-05 | SPRR1B |
| rs4845515 | 1 | 151270495 | t | g | 4.05 | 5.2E-05 | SPRR1B |
| rs4845336 | 1 | 151270477 | t | c | 4.05 | 5.2E-05 | SPRR1B |
| rs13253966 | 8 | 43177542 | t | c | -4.04 | 5.3E-05 | HGSNAT |
| rs892762 | 3 | 165585347 | t | c | 4.04 | 5.3E-05 | SI |
| rs4821419 | 22 | 34323729 | a | c | -4.04 | 5.3E-05 | MB |
| rs13328676 | 1 | 201644182 | c | g | 4.04 | 5.4E-05 | FMOD |
| rs441229 | 1 | 151281683 | a | c | -4.04 | 5.4E-05 | SPRR2D |
| rs1415969 | 1 | 151273072 | t | g | 4.04 | 5.4E-05 | SPRR1B |
| rs7203576 | 16 | 81745773 | a | g | -4.04 | 5.4E-05 | CDH13 |
| rs13271165 | 8 | 43185345 | t | c | 4.04 | 5.4E-05 | HGSNAT |
| rs9637776 | 5 | 31315062 | a | g | 4.04 | 5.4E-05 | CDH6 |
| rs1521247 | 2 | 12958863 | c | g | -4.04 | 5.5E-05 | TRIB2 |
| rs3819154 | 21 | 37256632 | c | g | 4.03 | 5.5E-05 | HLCS |
| rs368912 | 1 | 151282828 | c | g | -4.03 | 5.5E-05 | SPRR2D |
| rs6719434 | 2 | 12780356 | t | c | -4.03 | 5.5E-05 | TRIB2 |
| rs12234734 | 7 | 95017449 | t | c | 4.03 | 5.5E-05 | ASB4 |
| rs7623591 | 3 | 165594450 | a | g | -4.03 | 5.5E-05 | SI |
| rs11577955 | 1 | 151386143 | a | g | 4.03 | 5.6E-05 | SPRR2G |
| rs6945696 | 7 | 89891600 | t | c | 4.03 | 5.6E-05 | CLDN12 |
| rs189687 | 5 | 128120719 | a | t | 4.03 | 5.7E-05 | SLC27A6 |
| rs9683743 | 4 | 39379589 | t | g | 4.03 | 5.7E-05 | HIP2 |
| rs6432343 | 2 | 12957397 | a | g | -4.03 | 5.7E-05 | TRIB2 |
| rs6767488 | 3 | 165600556 | t | c | 4.02 | 5.7E-05 | SI |
| rs9824997 | 3 | 165599759 | a | g | 4.02 | 5.7E-05 | SI |
| rs721975 | 3 | 165583839 | t | g | 4.02 | 5.8E-05 | SI |
| rs1933382 | 1 | 151269919 | t | g | -4.02 | 5.8E-05 | SPRR1B |
| rs7638502 | 3 | 165604668 | a | g | -4.02 | 5.8E-05 | SI |
| rs1415966 | 1 | 151269488 | a | g | -4.02 | 5.8E-05 | SPRR1B |
| rs6762010 | 3 | 165581417 | t | g | 4.02 | 5.8E-05 | SI |
| rs13271956 | 8 | 43098985 | t | c | 4.02 | 5.8E-05 | FLJ23356 |
| rs1469215 | 2 | 12803771 | t | g | 4.02 | 5.8E-05 | TRIB2 |
| rs162188 | 6 | 134268916 | a | g | 4.02 | 5.8E-05 | TCF21 |
| rs4974948 | 4 | 39429031 | t | g | -4.02 | 5.9E-05 | HIP2 |
| rs1415965 | 1 | 151269374 | a | g | 4.02 | 5.9E-05 | SPRR1B |
| rs1467348 | 12 | 130140594 | c | g | -4.02 | 5.9E-05 | GPR133 |
| rs10513613 | 3 | 165606556 | t | c | -4.02 | 5.9E-05 | SI |
| rs162187 | 6 | 134268014 | t | c | 4.02 | 5.9E-05 | TCF21 |
| rs9290232 | 3 | 165607917 | t | c | 4.01 | 6.0E-05 | SI |
| rs10788855 | 1 | 151268983 | a | t | -4.01 | 6.0E-05 | SPRR1B |
| rs4490578 | 5 | 19301858 | a | g | -4.01 | 6.0E-05 | CDH18 |
| rs11205174 | 1 | 151267854 | a | g | -4.01 | 6.0E-05 | SPRR1B |
| rs11205175 | 1 | 151267920 | a | c | -4.01 | 6.0E-05 | SPRR1B |
| rs3781290 | 10 | 104234938 | c | g | -4.01 | 6.1E-05 | ACTR1A |
| rs9840047 | 3 | 165608266 | t | g | 4.01 | 6.1E-05 | SI |
| rs892764 | 3 | 165584473 | a | c | -4.01 | 6.1E-05 | SI |
| rs10888526 | 1 | 151266935 | t | g | 4.01 | 6.1E-05 | SPRR1B |
| rs6813345 | 4 | 38257918 | c | g | 4.01 | 6.1E-05 | KLF3 |
| rs16848141 | 3 | 165606061 | a | g | 4.01 | 6.1E-05 | SI |
| rs1821575 | 3 | 165584968 | a | g | 4.01 | 6.2E-05 | SI |
| rs162183 | 6 | 134267528 | a | c | -4.01 | 6.2E-05 | TCF21 |
| rs6683514 | 1 | 151251021 | a | g | -4.01 | 6.2E-05 | SPRR3 |
| rs7637477 | 3 | 165609496 | a | g | -4.00 | 6.2E-05 | SI |
| rs1879543 | 2 | 12942282 | t | c | 4.00 | 6.3E-05 | TRIB2 |
| rs2059428 | 2 | 12810380 | t | c | 4.00 | 6.4E-05 | TRIB2 |
| rs7614257 | 3 | 165615329 | t | c | -4.00 | 6.4E-05 | SI |
| rs2888089 | 2 | 212927526 | a | c | 4.00 | 6.4E-05 | ERBB4 |
| rs4477031 | 8 | 48136000 | t | c | 4.00 | 6.5E-05 | KIAA0146 |
| rs12543695 | 8 | 48136481 | a | g | 3.99 | 6.5E-05 | KIAA0146 |
| rs3788512 | 22 | 34332366 | t | c | 3.99 | 6.5E-05 | MB |
| rs6991259 | 8 | 48133869 | a | t | -3.99 | 6.5E-05 | KIAA0146 |
| rs7004078 | 8 | 48133356 | a | g | 3.99 | 6.6E-05 | KIAA0146 |
| rs11912323 | 22 | 34302725 | t | c | -3.99 | 6.6E-05 | RASD2 |
| rs2042566 | 2 | 12796119 | t | c | 3.99 | 6.7E-05 | TRIB2 |
| rs13050469 | 21 | 42067716 | t | c | -3.99 | 6.7E-05 | RIPK4 |
| rs328455 | 6 | 134263946 | t | c | -3.99 | 6.8E-05 | TCF21 |
| rs7016791 | 8 | 48113856 | a | g | -3.99 | 6.8E-05 | KIAA0146 |
| rs10212606 | 3 | 165615870 | t | c | 3.98 | 6.8E-05 | SI |
| rs13048349 | 21 | 42067528 | t | g | 3.98 | 6.8E-05 | RIPK4 |
| rs779343 | 2 | 12966550 | t | c | 3.98 | 6.8E-05 | TRIB2 |
| rs13273902 | 8 | 48122853 | a | t | -3.98 | 6.8E-05 | KIAA0146 |
| rs7618193 | 3 | 165617477 | a | g | -3.98 | 6.8E-05 | SI |
| rs7687557 | 4 | 39445655 | a | c | 3.98 | 6.9E-05 | HIP2 |
| rs7694987 | 4 | 95911500 | a | g | 3.98 | 6.9E-05 | BMPR1B |
| rs2042567 | 2 | 12796093 | t | c | -3.98 | 6.9E-05 | TRIB2 |
| rs162186 | 6 | 134267948 | t | c | 3.98 | 7.0E-05 | TCF21 |
| rs7666429 | 4 | 87038739 | a | g | -3.98 | 7.0E-05 | ARHGAP24 |
| rs13251524 | 8 | 42939665 | a | g | 3.98 | 7.0E-05 | HOOK3 |
| rs6814315 | 4 | 39469780 | t | g | 3.97 | 7.1E-05 | HIP2 |
| rs1052654 | 8 | 43004512 | a | c | -3.97 | 7.2E-05 | HOOK3 |
| rs1920735 | 12 | 89982350 | a | g | 3.97 | 7.2E-05 | KERA |
| rs12498985 | 4 | 126144883 | t | c | 3.97 | 7.3E-05 | ANKRD50 |
| rs4041402 | 1 | 151214021 | t | c | -3.97 | 7.3E-05 | SPRR4 |
| rs11205177 | 1 | 151269126 | a | g | 3.97 | 7.3E-05 | SPRR1B |
| rs3816090 | 3 | 165618651 | t | c | 3.96 | 7.4E-05 | SI |
| rs7617240 | 3 | 165571027 | t | c | 3.96 | 7.4E-05 | SI |
| rs4855220 | 3 | 165576703 | t | c | 3.96 | 7.4E-05 | SI |
| rs1510580 | 4 | 87036129 | t | c | 3.96 | 7.5E-05 | ARHGAP24 |
| rs1708471 | 3 | 165663685 | a | g | 3.96 | 7.5E-05 | SI |
| rs10786685 | 10 | 104313093 | a | g | -3.96 | 7.5E-05 | SUFU |
| rs1016510 | 4 | 87038401 | a | c | -3.96 | 7.5E-05 | ARHGAP24 |
| rs2304377 | 3 | 165618866 | a | g | -3.96 | 7.5E-05 | SI |
| rs10888524 | 1 | 151266749 | a | g | -3.96 | 7.6E-05 | SPRR1B |
| rs10888525 | 1 | 151266839 | a | g | 3.96 | 7.6E-05 | SPRR1B |
| rs9578831 | 13 | 24652557 | t | c | 3.96 | 7.6E-05 | FAM123A |
| rs4845503 | 1 | 151226563 | a | g | -3.96 | 7.7E-05 | SPRR1A |
| rs10788853 | 1 | 151268855 | t | c | 3.95 | 7.7E-05 | SPRR1B |
| rs2279510 | 10 | 98853179 | t | c | 3.95 | 7.8E-05 | SLIT1 |
| rs6851522 | 4 | 87043247 | a | c | -3.95 | 7.9E-05 | ARHGAP24 |
| rs783586 | 3 | 165654173 | a | g | -3.95 | 7.9E-05 | SI |
| rs950612 | 2 | 12907485 | c | g | 3.95 | 8.0E-05 | TRIB2 |
| rs17465721 | 2 | 12880269 | t | c | 3.95 | 8.0E-05 | TRIB2 |
| rs5755786 | 22 | 34327146 | a | t | -3.94 | 8.0E-05 | MB |
| rs5750126 | 22 | 34327333 | a | g | -3.94 | 8.1E-05 | MB |
| rs4263408 | 4 | 39461671 | t | c | 3.94 | 8.1E-05 | HIP2 |
| rs4917756 | 10 | 98865398 | a | g | 3.94 | 8.1E-05 | SLIT1 |
| rs4344931 | 2 | 241467200 | a | c | -3.94 | 8.1E-05 | AGXT |
| rs11158820 | 14 | 69417101 | a | g | -3.94 | 8.1E-05 | SMOC1 |
| rs11769121 | 7 | 157077839 | a | t | -3.94 | 8.1E-05 | PTPRN2 |
| rs10086987 | 8 | 48208696 | c | g | 3.94 | 8.2E-05 | KIAA0146 |
| rs4515742 | 1 | 151252253 | a | g | -3.94 | 8.2E-05 | SPRR3 |
| rs1490688 | 10 | 98850464 | t | c | 3.94 | 8.2E-05 | SLIT1 |
| rs1394782 | 2 | 212909165 | a | g | 3.93 | 8.3E-05 | ERBB4 |
| rs2035550 | 5 | 96861245 | t | g | 3.93 | 8.4E-05 | RIOK2 |
| rs1510582 | 4 | 87043546 | t | c | 3.93 | 8.5E-05 | ARHGAP24 |
| rs4495437 | 8 | 48205969 | t | g | 3.93 | 8.5E-05 | KIAA0146 |
| rs4821424 | 22 | 34331806 | a | g | -3.93 | 8.6E-05 | MB |
| rs6893784 | 5 | 96871109 | a | g | 3.93 | 8.6E-05 | RIOK2 |
| rs1327268 | 6 | 51280428 | c | g | -3.93 | 8.6E-05 | PKHD1 |
| rs16994660 | 21 | 37275424 | t | c | 3.93 | 8.6E-05 | HLCS |
| rs1505356 | 2 | 212913246 | a | c | 3.93 | 8.7E-05 | ERBB4 |
| rs2521285 | 11 | 2298412 | a | g | -3.93 | 8.7E-05 | TSPAN32 |
| rs3213150 | 20 | 31735862 | a | g | -3.92 | 8.7E-05 | E2F1 |
| rs1890285 | 1 | 151282020 | t | c | -3.92 | 8.7E-05 | SPRR2D |
| rs12578934 | 12 | 89990627 | a | g | -3.92 | 8.7E-05 | KERA |
| rs10191751 | 2 | 12879766 | a | g | -3.92 | 8.8E-05 | TRIB2 |
| rs1357121 | 11 | 75627443 | a | g | 3.92 | 8.8E-05 | WNT11 |
| rs12826445 | 12 | 10887583 | a | g | 3.92 | 9.0E-05 | PRR4 |
| rs12606601 | 18 | 23541951 | a | g | -3.91 | 9.3E-05 | CDH2 |
| rs4387369 | 11 | 75624150 | t | g | -3.91 | 9.3E-05 | WNT11 |
| rs1876048 | 2 | 212914094 | a | g | 3.91 | 9.4E-05 | ERBB4 |
| rs344030 | 3 | 157947423 | a | c | 3.91 | 9.4E-05 | TIPARP |
| rs7829867 | 8 | 42889108 | t | c | -3.91 | 9.4E-05 | HOOK3 |
| rs2135157 | 2 | 212914547 | t | c | 3.91 | 9.4E-05 | ERBB4 |
| rs12238375 | 9 | 83386335 | t | c | 3.90 | 9.5E-05 | TLE1 |
| rs3936203 | 2 | 241483327 | a | g | -3.90 | 9.5E-05 | C2orf54 |
| rs4919069 | 10 | 98851074 | t | c | 3.90 | 9.6E-05 | SLIT1 |
| rs4311055 | 2 | 241469690 | t | c | -3.90 | 9.6E-05 | AGXT |
| rs2038068 | 6 | 35482439 | a | g | -3.90 | 9.6E-05 | PPARD |
| rs209665 | 20 | 31592550 | t | g | 3.90 | 9.7E-05 | CBFA2T2 |
| rs7110233 | 11 | 80059135 | a | t | 3.90 | 9.8E-05 | ODZ4 |
| rs1800649 | 2 | 97631289 | t | c | 3.89 | 9.9E-05 | COX5B |
| rs4974230 | 3 | 60755953 | t | c | 3.89 | 9.9E-05 | FHIT |
