# Supplementary material for: Genome-Wide Association of Pericardial Fat Identifies a Unique Locus for Ectopic Fat
Source: PLoS Genet. 2012 May 10;8(5):e1002705. doi: 10.1371/journal.pgen.1002705 (PMC3349742; doi:10.1371/journal.pgen.1002705)
Supplement: Supplementary file 3 [file pgen.1002705.s004.doc]

Study	Array type	Genotype calling	QC filters for genotyped SNPs used for imputation	No of SNPs used for imputation	Imputation	Imputation Backbone for phased CEU haplotypes (NCBI build)	Filtering of imputed genotypes	Data management and statistical analysis
**Framingham Heart Study**	Affymetrix 500K Affymetrix 50K supplemental	Affymetrix	pHWE<1e-6, call rate<97%, mishap p<1e-9, MAF<0.01, Mendelian errors>100, SNPs not in Hapmap or strandedness issues merging with Hapmap	378,163	MACH version 1.0.15	HapMap release 22 (build 36)	none	R, linear mixed effect models and GEE models, robust variance option to account for relatedness
**MESA**	Affymetrix Genome-Wide Human SNP Array 6.0	Affymetrix	call rate<95%, MAF<0.01	861,124	IMPUTE version 2.1.0	HapMap release 22 (build 36)	none	R
