# Supplementary material for: Genome-Wide Association of Pericardial Fat Identifies a Unique Locus for Ectopic Fat
Source: PLoS Genet. 2012 May 10;8(5):e1002705. doi: 10.1371/journal.pgen.1002705 (PMC3349742; doi:10.1371/journal.pgen.1002705)
Supplement: Supplementary file 4 [file pgen.1002705.s006.doc]

SNP	Gene	Risk Allele	OR from CARDIoGRAM	p-value from CARDIoGRAM	Coded Allele, Pericardial Fat Meta-analysis	Z-score	p-value	q-values
rs11206510	PCSK9	0.82 (T)	1.08 (1.05–1.11)	9.10 × 10−8	t	0.460	0.646	0.993
rs599839	SORT1	0.78 (A)	1.11 (1.08–1.15)	2.89 × 10−10	a	0.139	0.890	0.993
rs17465637	MIA3	0.74 (C)	1.14 (1.09–1.20)	1.36 × 10−8	a	-0.570	0.569	0.993
rs6725887	WDR12	0.15 (C)	1.14 (1.09–1.19)	1.12 × 10−9	t	0.107	0.915	0.993
rs2306374	MRAS	0.18 (C)	1.12 (1.07–1.16)	3.34 × 10−8	t	-1.481	0.139	0.555
rs12526453	PHACTR1	0.67 (C)	1.10 (1.06–1.13)	1.15 × 10−9	c	2.353	0.019	0.123
rs3798220	LPA	0.02 (C)	1.51 (1.33–1.70)	3.00 × 10−11	NA	NA	NA	NA
rs4977574	CDKN2A,CDKN2B	0.46 (G)	1.29 (1.23–1.36)	1.35 × 10−22	a	-1.664	0.096	0.461
rs1746048	CXCL12	0.87 (C)	1.09 (1.07–1.13)	2.93 × 10−10	t	0.402	0.688	0.993
rs3184504	SH2B3	0.44 (T)	1.07 (1.04–1.10)	6.35 × 10−6	t	0.009	0.993	0.993
rs1122608	LDLR	0.77 (G)	1.14 (1.09–1.18)	9.73 × 10−10	t	0.802	0.423	0.993
rs9982601	MRPS6	0.15 (T)	1.18 (1.12–1.24)	4.22 × 10−10	t	0.460	0.645	0.993
rs17114036	*PPAP2B*	0.91 (A)	1.17 (1.13–1.22)	3.81 × 10−19	a	2.317	0.020	0.123
rs17609940	*ANKS1A*	0.75 (G)	1.07 (1.05–1.10)	1.36 × 10−8	c	0.887	0.375	0.993
rs12190287	*TCF21*	0.62 (C)	1.08 (1.06–1.10)	1.07 × 10−12	c	3.102	0.0019	0.046
rs11556924	*ZC3HC1*	0.62 (C)	1.09 (1.07–1.12)	9.18 × 10−18	t	-0.931	0.352	0.993
rs579459	*ABO*	0.21 (C)	1.10 (1.07–1.13)	4.08 × 10−14	t	-0.694	0.488	0.993
rs12413409	*CYP17A1, CNNM2, NT5C2*	0.89 (G)	1.12 (1.08–1.16)	1.03 × 10−9	a	0.194	0.846	0.993
rs964184	*ZNF259, APOA5-A4-C3-A1*	0.13 (G)	1.13 (1.10–1.16)	1.02 × 10−17	c	2.735	0.006	0.075
rs4773144	*COL4A1, COL4A2*	0.44 (G)	1.07 (1.05–1.09)	3.84 × 10−9	a	-0.526	0.599	0.993
rs2895811	*HHIPL1*	0.43 (C)	1.07 (1.05–1.10)	1.14 × 10−10	t	0.035	0.972	0.993
rs3825807	*ADAMTS7*	0.57 (A)	1.08 (1.06–1.10)	1.07 × 10−12	a	0.275	0.784	0.993
rs216172	*SMG6, SRR*	0.37 (C)	1.07 (1.05–1.09)	1.15 × 10−9	c	0.970	0.332	0.993
rs12936587	*RASD1, SMCR3, PEMT*	0.56 (G)	1.07 (1.05–1.09)	4.45 × 10−10	a	0.009	0.993	0.993
rs46522	*UBE2Z, GIP, ATP5G1, SNF8*	0.53 (T)	1.06 (1.04–1.08)	1.81 × 10−8	t	0.131	0.896	0.993
