# Supplementary material for: Genome-Wide Association of Pericardial Fat Identifies a Unique Locus for Ectopic Fat
Source: PLoS Genet. 2012 May 10;8(5):e1002705. doi: 10.1371/journal.pgen.1002705 (PMC3349742; doi:10.1371/journal.pgen.1002705)
Supplement: Supplementary file 5 [file pgen.1002705.s007.doc]

SNP	Chromosome	Nearby Genes	Coded Allele	Z-score	P-value
rs984222	1	*TBX15-WARS2*	G	1.82	0.07
rs1011731	1	*DNM3-PIGC*	G	1.77	0.08
rs4846567	1	*LYPLAL1*	G	1.64	0.10
rs10195252	2	*GRB14*	T	-0.24	0.81
rs6784615	3	*NISCH-STAB1*	T	-0.98	0.33
rs6795735	3	*ADAMTS9*	C	0.07	0.95
rs6861681	5	*CPEB4*	A	3.05	0.002
rs1294421	6	*LY86*	G	-0.09	0.93
rs6905288	6	*VEGFA*	A	1.11	0.27
rs9491696	6	*RSPO3*	G	-0.66	0.51
rs1055144	7	*NFE2L3*	T	1.08	0.28
rs718314	12	*ITPR2-SSPN*	G	-1.19	0.23
rs1443512	12	*HOXC13*	A	0.11	0.91
rs4823006	22	*ZNRF3-KREMEN1*	A	0.18	0.86
