# Supplementary material for: Genome-Wide Association of Pericardial Fat Identifies a Unique Locus for Ectopic Fat
Source: PLoS Genet. 2012 May 10;8(5):e1002705. doi: 10.1371/journal.pgen.1002705 (PMC3349742; doi:10.1371/journal.pgen.1002705)
Supplement: Supplementary file 6 [file pgen.1002705.s008.doc]

SNP	Chr	Closest Gene	Coded allele	Z	P-value
rs9816226	3	*ETV5*	T	0.25	0.80
rs2287019	19	*GIPR*	C	0.76	0.45
rs1558902	16	*FTO*	A	2.15	0.03
rs11847697	14	*PRKD1*	T	-0.55	0.58
rs7359397	16	*SH2B1*	T	0.31	0.75
rs2241423	15	*MAP2K5*	G	-0.50	0.61
rs7138803	12	*FAIM2*	A	0.75	0.45
rs10150332	14	*NRXN3*	C	1.52	0.13
rs12444979	16	*GPRC5B*	C	-0.19	0.85
rs887912	2	*FANCL*	T	-0.72	0.47
rs2112347	5	*FLJ35779*	T	-0.01	0.99
rs2815752	1	*NEGR1*	A	1.66	0.10
rs4836133	5	*ZNF608*	A	1.37	0.17
rs571312	18	*MC4R*	A	1.18	0.24
rs3810291	19	*TMEM160*	A	-1.62	0.10
rs4929949	11	*RPL27A*	C	-0.07	0.94
rs713586	2	*RBJ, ADCY3*	C	-0.44	0.66
rs2890652	2	*LRP1B*	C	-1.00	0.32
rs987237	6	*TFAP2B*	G	-0.24	0.81
rs10968576	9	*LINGO2*	G	0.38	0.70
rs13107325	4	*SLC39A8*	T	0.18	0.85
rs3817334	11	*MTCH2*	T	-1.09	0.28
rs206936	6	*NUDT3*	G	0.52	0.60
rs2867125	2	*TMEM18*	C	0.73	0.47
rs543874	1	*SEC16B*	G	-1.04	0.30
rs1555543	1	*PTBP2*	C	0.90	0.37
rs1514175	1	*TNNI3K*	A	-0.72	0.47
rs10938397	4	*GNPDA2*	G	0.28	0.78
rs29941	19	*KCTD15*	G	0.09	0.93
rs13078807	3	*CADM2*	G	-0.07	0.94
rs10767664	11	*BDNF*	A	-0.66	0.51
rs4771122	13	*MTIF3*	G	-1.74	0.08
